# The *Coxiella burnetii* Dot/Icm System Delivers a Unique Repertoire of Type IV Effectors into Host Cells and Is Required for Intracellular Replication

**DOI:** 10.1371/journal.ppat.1002056

**Published:** 2011-05-26

**Authors:** Kimberly L. Carey, Hayley J. Newton, Anja Lührmann, Craig R. Roy

**Affiliations:** 1 Section of Microbial Pathogenesis, Yale University School of Medicine, New Haven, Connecticut, United States of America; 2 Microbiology Institute, University Clinic Erlangen, Friedrich-Alexander University of Erlangen-Nuremberg, Germany; Texas Medical Center, United States of America

## Abstract

*Coxiella burnetii*, the causative agent of human Q fever, is an intracellular pathogen that replicates in an acidified vacuole derived from the host lysosomal network. This pathogen encodes a Dot/Icm type IV secretion system that delivers bacterial proteins called effectors to the host cytosol. To identify new effector proteins, the functionally analogous *Legionella pneumophila* Dot/Icm system was used in a genetic screen to identify fragments of *C. burnetii* genomic DNA that when fused to an adenylate cyclase reporter were capable of directing Dot/Icm-dependent translocation of the fusion protein into mammalian host cells. This screen identified Dot/Icm effectors that were proteins unique to *C. burnetii*, having no overall sequence homology with *L. pneumophila* Dot/Icm effectors. A comparison of *C. burnetii* genome sequences from different isolates revealed diversity in the size and distribution of the genes encoding many of these effectors. Studies examining the localization and function of effectors in eukaryotic cells provided evidence that several of these proteins have an affinity for specific host organelles and can disrupt cellular functions. The identification of a transposon insertion mutation that disrupts the *dot/icm* locus was used to validate that this apparatus was essential for translocation of effectors. Importantly, this *C. burnetii* Dot/Icm-deficient mutant was found to be defective for intracellular replication. Thus, these data indicate that *C. burnetii* encodes a unique subset of bacterial effector proteins translocated into host cells by the Dot/Icm apparatus, and that the cumulative activities exerted by these effectors enables *C. burnetii* to successfully establish a niche inside mammalian cells that supports intracellular replication.

## Author Summary

*Coxiella burnetii* is a Gram-negative intracellular bacterium that can cause the human disease Q fever. A type IV secretion system in *C. burnetii* called Dot/Icm is functionally similar to the Dot/Icm system of *Legionella pneumophila*. Here we used *L. pneumophila* to screen a *C. burnetii* library for genes encoding effector proteins. We identified 18 effectors that are unique to *C. burnetii* and show that when they are expressed in eukaryotic cells they localize to specific compartments and can mediate changes in host cell physiology. Comparative genomic analysis revealed plasticity among these novel effector proteins that could be related to the different manifestations of disease exhibited by these clinical isolates of *C. burnetii*. A transposon insertion mutation in the *dot/icm* locus revealed for the first time that type IV secretion is essential for *C. burnetii* replication inside mammalian host cells and that the delivery of effectors requires Dot/Icm function. Thus, this study conclusively shows that the *C. burnetii* Dot/Icm system is an essential determinant for intracellular replication, and identifies a repertoire of unique effector proteins with novel functions delivered by this system that could be important for disease phenotypes.

## Introduction

*Coxiella burnetii*, a Gram-negative facultative intracellular bacterium, is the causative agent of human Q fever, a worldwide zoonosis predominantly linked to exposure to domesticated livestock. Q fever typically manifests as an acute flu-like illness, however, chronic Q fever can also develop, typically in immunocompromised individuals [1].

Fundamental to the pathogenesis of *C. burnetii* is the capacity to replicate within a specialized vacuole that is derived from host lysosomes. *C. burnetii* requires the low pH environment of the lysosome to convert from the environmentally-resistant small cell variant that is the infectious form of the bacterium to a large cell variant that represents the replicative form of the bacterium [2], [3]. The *Coxiella*-containing vacuole (CCV) retains the degradative capacity of a lysosome [4], and is decorated by the host lipid raft proteins flotilin 1 and 2 and the autophagosome marker LC3 [5], [6]. The CCV also undergoes expansion, often occupying the majority of the host cytoplasm, through robust homotypic fusion with endolysosomal vesicles [7]. Importantly, the formation and development of this fusogenic CCV is dependent on bacterial protein synthesis [7], implying that *C. burnetii* directs formation of the vacuole in which it resides.

*C. burnetii* proteins important for establishment of this specialized vacuole are predicted to be translocated into the cytosol of the host cell by the Dot/Icm type IV secretion system. This secretion system has both sequence homology and functional similarity to the Dot/Icm apparatus of *Legionella pneumophila* [8]–[10], which is involved in manipulating cellular functions in the protozoan hosts that *L. pneumophila* has coevolved with in nature [11], and also in mammalian cells that represent accidental hosts for this bacterium [12]–[14]. Within these evolutionarily diverse phagocytic host cells, the *L. pneumophila* Dot/Icm system is essential for establishment of a unique endoplasmic reticulum-derived vacuole that enables intracellular survival and replication of this pathogen [15]–[17]. It is estimated that *L. pneumophila* is capable of translocating over 200 different proteins using the Dot/Icm system [18], [19]. Loss of a single effector protein in *L. pneumophila* does not typically diminish intracellular replication, indicating a degree of functional redundancy among the effectors that is not resolved through standard approaches involving forward genetic analysis. Defined aspects of the *L. pneumophila* vacuole morphology, however, can be linked to specific Dot/Icm effector proteins. For example, the effector DrrA (SidM) has a specific role in manipulating the function of the host GTPase Rab1 and promoting the localization of Rab1 to vacuoles containing *L. pneumophila* [20]–[22]. Similarly, the effector RalF recruits the host GTPase Arf1 to vacuoles, and *ralF* mutant bacteria occupy vacuoles that fail to recruit Arf1 to their limiting membrane [23].

Although proteins that have limited regions of homology with *L. pneumophila* effectors are encoded by *C. burnetii*, this intracellular pathogen does not appear to produce *bone fide* homologues of *L. pneumophila* Dot/Icm effector proteins. This suggests that these pathogens possess unique effector repertoires that could reflect the divergent pathways that have resulted in these organisms occupying unique replicative niches within evolutionarily diverse eukaryotic hosts. *L. pneumophila* was used previously as a surrogate to demonstrate several *C. burnetii* Ank proteins containing ankyrin repeat homology domains are translocated into mammalian hosts by a Dot/Icm-dependent mechanism [24], [25]. Comparative genomics revealed a high degree of variation of these Ank proteins among different isolates of *C. burnetii*, with mutations rendering many Ank coding regions as putative pseudogenes [25]. One of the few Ank proteins conserved among the sequenced *C. burnetii* isolates is AnkG, which has been demonstrated to mediate potent anti-apoptotic activity when translocated into mammalian host cells by the Dot/Icm system [26]. Given the diverse repertoire of Dot/Icm effectors encoded by *L. pneumophila*, there should be many novel effectors encoded by *C. burnetii* in addition to the Ank proteins.

The goal of this study was to identify new effectors from *C. burnetii* by conducting an unbiased screen for type IV secretion signals capable of delivering the calmodulin-dependent adenylate cyclase protein into host cells by the Dot/Icm system, and determine whether the delivery of effectors by *C. burnetii* is important for host cell infection.

## Results

### A screen for type IV translocation signals identifies novel *C. burnetii* effectors

Most Dot/Icm effectors contain a translocation signal recognized by the type IV apparatus, which is typically located near the carboxyl terminal region of the protein [18], [25], [27]. Thus, an unbiased genetic screen was designed to identify *C. burnetii* proteins having Dot/Icm-dependent translocation signals. A library was constructed by inserting random fragments of genomic DNA from *C. burnetii* RSA493 Nine Mile phase II (NM) downstream of a plasmid-encoded adenylate cyclase (Cya) enzyme that would serve as a reporter for translocation of proteins into the cytosol of host cells by the Dot/Icm system. The *C. burnetii* genomic DNA fragments were generated by limited digestion with the restriction enzyme *SauIIIA1* and ligation of the resulting DNA fragments downstream of a *cya* reporter in the plasmid pEC33. The resulting plasmid library was introduced into *L. pneumophila* to screen for *C. burnetii* genes containing a type IV translocation signal that when fused in frame with *cya* would result in a hybrid protein delivered into mammalian host cells by the Dot/Icm system.

Because the possibility of a single plasmid in the library having a DNA fragment inserted in the correct orientation and in frame with *cya* was predicted to be roughly one in six, the decision was made to screen a pool of bacteria containing different plasmid clones rather than initially screening individual clones from the library. To determine the feasibility of identifying a single translocated effector in pools of transformants, *L. pneumophila* expressing Cya-RalF, a fusion protein known to be efficiently translocated by the Dot/Icm system [23], [27], and *L. pneumophila* producing Cya alone, were mixed at defined ratios. Infection at a ratio of bacteria producing Cya-RalF to bacteria producing the control Cya of 1∶50 reproducibly led to an increase in host cell cAMP levels of at least 2.5-fold over background levels (Figure S1). These data indicated that it should be possible to detect a single positive clone with a type IV-dependent translocation signal fused to Cya in a mixed pool containing roughly 50 negative clones. Thus, the library of clones was distributed into pools that contained an estimated 50 different transformants harboring a randomly inserted *C. burnetii* gene downstream of *cya* (Figure S2). Mammalian CHO FcγRII cells [28] were infected with the pools of *L. pneumophila* containing Cya fused randomly to *C. burnetii* gene products. Screening identified pools that resulted in a significant increase in host cAMP levels above background, and these pools were further analyzed to isolate individual clones with an in-frame fusion between *cya* and a *C. burnetii* gene having a functional Dot/Icm translocation signal.

A total of 22-positive clones were identified from the 506 different pools tested in the translocation assay, which would theoretically represent the screening of 4200 in-frame fusions. Plasmids were isolated from the positive clones and DNA sequencing was used to identify the predicted *C. burnetii* protein fused to Cya. This analysis resulted in the identification of 11 potential Dot/Icm effector proteins (Table S1), including the previously characterized Dot/Icm effector AnkG (CBU0781) [24]–[26].

**Figure 1:**
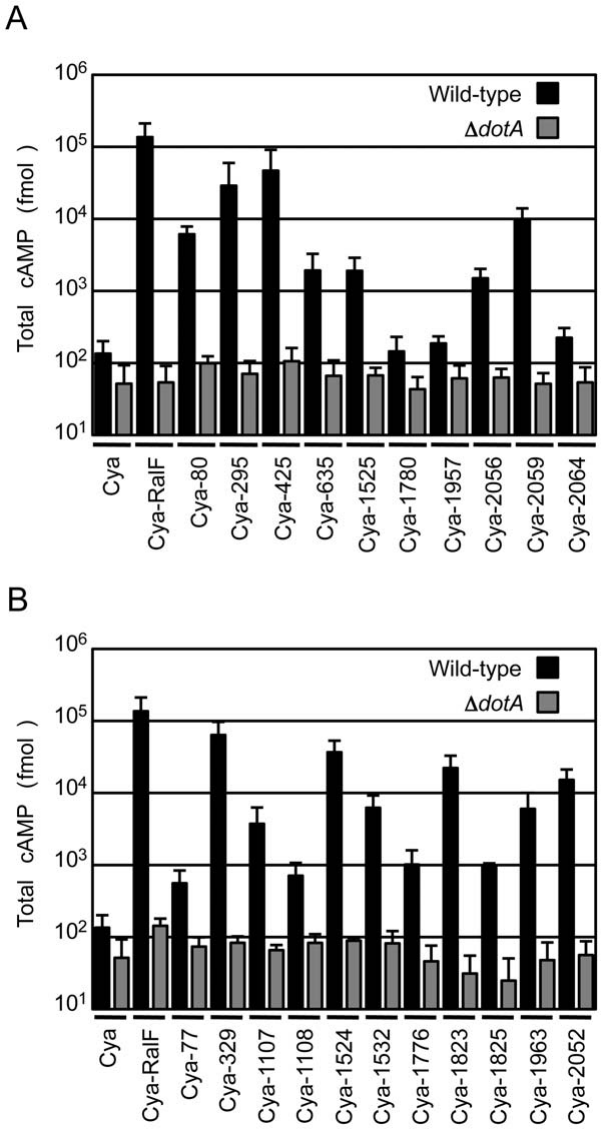
Dot/Icm-dependent translocation of *C. burnetii* proteins by *L. pneumophila*. CHO-FcγRII cells were infected with Dot/Icm-sufficient LP01 strain of *L. pneumophila* (black) or the isogenic Δ*dotA* mutant (grey) expressing Cya fusions to the indicated *C. burnetii* proteins. (A) Fusions to Cya of the indicated full-length derivatives of *C. burnetii* NM proteins identified in the genetic screen were tested for translocation (B). Fusions to Cya of the indicated full-length derivatives of *C. burnetii* NM proteins identified based on homology or proximity to proteins identified in the screen were tested for translocation. Cya indicates empty vector control. Cya-RalF was used as a positive control. After 1 h, host cells were lysed and cAMP was extracted. Total cAMP levels resulting from translocation of protein fusions were quantified using an enzyme-immunoassay system, and are shown in fmol. Results represent average values +/− SD of experiments performed in triplicate.

To validate that the proteins identified in the screen were *bone fide* type IV effectors, the predicted full-length open reading frames for each of the 10 new proteins identified in the screen were amplified using genomic DNA from the *C. burnetii* NM strain, and the resulting full-length NM gene was fused to the C-terminus of Cya. Plasmids encoding a putative full-length effector fused to Cya were introduced into a strain of *L. pneumophila* having a functional Dot/Icm system and a Δ*dotA* mutant strain with a non-functional type IV apparatus [15]. Seven of the 10 *C. burnetii* fusion proteins showed significant increases in cAMP levels compared to controls (Figure 1A). Importantly, translocation was observed to be dependent upon a functional Dot/Icm system as cAMP levels were unchanged after infection with the Δ*dotA* strain (Figure 1A). The three fusion proteins that did not demonstrate translocation; Cya-1780, Cya-1957 and Cya-2064 were still expressed at levels comparable to Cya and Cya-RalF in *L. pneumophila* (Figure S3).

Because genes encoding *L. pneumophila* effectors are often found in clusters at distinct chromosomal locations and can be duplicated to generate families of paralogous effectors [29], we investigated whether any of the *C. burnetii* genes in close proximity or with extensive sequence similarity to the identified effectors might also encode effectors. Thirty-seven additional *C. burnetii* genes that fit these criteria were tested for translocation by *L. pneumophila* (Table S2). Of the 37 genes tested, there were 11 genes encoding proteins translocated into host cells by a Dot/Icm-dependent mechanism (Figure 1B). Thus, this screen in total resulted in the identification of 18 different *C. burnetii* proteins that are effectors delivered into host cells by the *L. pneumophila* Dot/Icm apparatus.

### Genomic comparisons indicate plasticity among novel *C. burnetii* effectors

**Figure 2:**
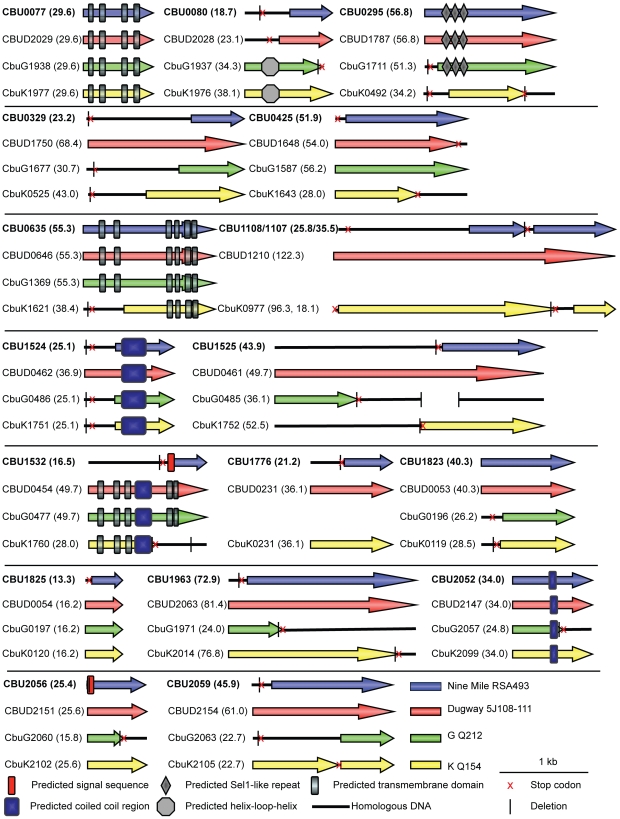
Domain analysis and genomic comparisons for *C. burnetii* effectors identified in this study. The schematic shows the eighteen NM effectors identified in this study represented in blue. Below each NM effector is a representation of the size of the homologous reading frame encoded by the genes in the other sequenced strains of *C. burnetii*. The size of the predicted proteins is represented in kDa in brackets following the gene designation. Black lines represent the presence of homologous DNA that does not encode an open reading frame due to small deletions (vertical black line) and stop codons (red cross). In cases where multiple deletions occur the first deletion, representing the site of the frameshift, is displayed. The locations of conserved domains in each protein identified in a SMART database search are indicated according to the key provided.

Sequence analysis revealed that none of the 18 *C. burnetii* Dot/Icm effectors have significant homology to proteins found in other organisms, demonstrating the unique nature of these proteins. CBU0077 from the strain NM RSA493 is highly conserved in the two different sequenced *C. burnetii* strains isolated from chronic Q-fever patients presenting with endocarditis (G Q212 and K Q154) and in the Dugway strain isolated from rodents [30], which does not appear to cause clinical disease. For the remaining effectors, there were polymorphisms in the coding regions that would indicate that these effectors have either been mutated or have not been acquired by one or more of the strains of *C. burnetii*. An alignment of these effectors in all sequenced strains is presented in Figure 2. A comparison of the NM genome to the genomes of the G, K and Dugway strains revealed nucleotide deletions or nonsense mutations in the NM genes that would predict the production of a shorter version of several effector proteins compared to homologues found in some of these other sequenced strains [31]. Interestingly, three of the NM effectors identified here, CBU1108, CBU1107 and CBU1776, are not present in the G Q212 strain (Figure 2). Thus, similar to Dot/Icm effectors in *L. pneumophila*, there is significant plasticity observed in the repertoire of effectors of *C. burnetii* when genomes from different isolates are compared [32]. Despite these polymorphisms, all of the genes encoding the NM Dot/Icm effectors identified here were transcribed during infection (Figure S4). Thus, the genes encoding truncated NM effectors are transcribed and the predicted translated products should be delivered into host cells during infection by the functional Dot/Icm translocation signal identified in the protein.

### Structural features and host cell localization phenotypes exhibited by *C. burnetii* effectors

Analysis of the *C. burnetii* effectors using the Simple Modular Architecture Research Tool (SMART) database [33] revealed domains in these proteins that could provide insight into potential functions (Figure 2). Identified features included predicted transmembrane domains (CBU0077/CBUD2029/CbuG1938/CbuK1977, CBU0635/CBUD0646/CbuG1369/CbuK1621, and CBUD0454/CbuG0477/CbuK1760), proteins with predicted coiled-coil regions (CBU1524/CBUD0462/CbuG0486/CbuK1751, CBUD0454/CbuG0477/CbuK1760, and CBU2052/CBUD2147/CbuG2057/CbuK2099), an effector with three Sel1-like repeats (SLRs; CBU0295/CBUD1787/CbuG1711) and a protein with a helix-loop-helix motif (CbuG1937/CbuK1976). SLR-containing proteins, found in eukaryotes and bacteria, are thought to mediate protein-protein interactions and have been found in several pathogenic bacteria. Of note, two of the *L. pneumophila* SLR-containing proteins, EnhC and LpnE, have been associated with virulence [34]–[36].

**Figure 3:**
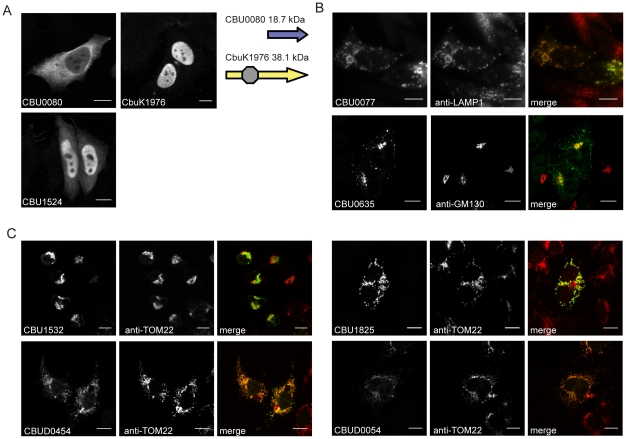
Effectors of *C. burnetii* show differential localization patterns when expressed in mammalian HeLa 229 cells. Immunofluorescence micrographs show the representative localization profiles of the indicated FLAG-tagged *C. burnetii* effectors (Green) and the indicated host organelles (Red) after ectopic production of the proteins in HeLa 229 cells. (A) CbuK1976 and CBU1524 show localization to the nucleus, but the NM protein CBU0080 is distributed throughout the cytoplasm. The schematic shows that CBU0080 does not contain the helix-loop-helix motif (octagon) found in the homolog CbuK1976, which could explain the differential localization phenotypes observed (B) Cells stained for FLAG-tagged CBU0077 and LAMP1 show colocalization of the effector on lysosome-derived organelles, and cells stained for CBU0635 and GM130 show distribution of the protein to a perinuclear region containing the Golgi apparatus. (C). Cells stained for FLAG-tagged effectors and the TOM22 protein show colocalization of the effectors on mitochondria. The NM protein CBU1825 and the homologous Dugway protein CBUD0054 showed similar localization patterns. The NM protein CBU1532 and the Dugway homolog CBUD0454 showed slightly different mitochondrial straining patterns, with CBU1532 production leading to cell rounding and mitochondria aggregation. Scale bars represent 10 µm.

To further characterize the identified Dot/Icm effectors, the products encoded by the NM genes and the longer derivatives encoded by the Dugway, G or K strains, were ectopically produced in HeLa 229 cells as proteins containing three tandem copies of the N-terminal FLAG epitope tag. All of the NM effectors demonstrated similar localization profiles as the homologous proteins isolated from the other *C. burnetii* strains, with the exception of CBU0080. The NM CBU0080 protein showed diffuse cytosolic distribution compared to the CbuK1976 protein, which localized to the nucleus (Figure 3A). The N-terminal 19.4 kDa region in CbuK1976 that is absent in CBU0080 encodes a predicted nuclear localization signal (NLS) containing the peptide KKRK in addition to a helix-loop-helix motif.

Most of the proteins localized to the host cell cytosol or diffusely throughout the cell when expressed in HeLa 229 cells (Table 1 and Figure S5). Several proteins showed enrichment in specific subcellular compartments as determined by colocalization with proteins that label these host organelles. Similar to CbuK1976, the CBU1524 protein localized to the nucleus when produced in mammalian cells (Figure 3A). Nuclear localization of CBU1524 may be mediated by a predicted seven residue NLS containing the peptide PKRTRVD that begins at amino acid position 182 in the protein. The protein CBU0077 resided primarily on lysosomes as demonstrated by colocalization with lysosomal-associated membrane protein 1 (LAMP1). The protein CBU0635 was present at a perinuclear position juxtaposed to the Golgi apparatus (Figure 3B). Two NM effector proteins, CBU1532 and CBU1825, along with the respective homologues from Dugway, CBUD0454 and CBUD0054, showed a specific affinity for mitochondria (Figure 3C). No defined mitochondrial targeting sequences were apparent in any of these effector proteins. Interestingly, CBU1532 appeared to affect the morphology of HeLa 229 cells, with many transfected cells having a rounded appearance with aggregated mitochondria. The Dugway protein, CBUD0454, did not mediate this morphological change when expressed in HeLa 229 cells, which might indicate that CBU1532 confers a dominant-negative effect that perturbs cellular functions modulated by CBUD0454. Similar localization patterns were observed in CHO-FcγRII cells and HEK 293 cells (data not shown), indicating that protein localization patterns are not cell-type specific.

**Table 1:** Characteristics of *C. burnetii* effector proteins.

NM effector	Localization^A^	SEAP Assay^B^	Yeast Doubling Time^C^
CBU0077	Lysosomal membrane	1.33±0.38	**5.39±0.29**
CBU0080	Cytoplasmic	0.94±0.32	4.21±0.29
CBU0295	Cytoplasmic	0.87±0.11	4.77±0.32
CBU0329	Cytoplasmic	0.95±0.22	4.85±0.35
CBU0425	Cytoplasmic	0.87±0.13	4.40±0.22
CBU0635	Golgi, vesicles	**0.49±0.17**	3.68±0.19
CBU1107	Cytoplasmic	1.07±0.21	4.38±0.16
CBU1108	Cytoplasmic	1.37±0.33	4.88±0.30
CBU1524	Nucleus	1.09±0.30	**5.90±0.39**
CBU1525	Cytoplasmic	1.18±0.36	**5.10±0.16**
CBU1532	Mitochondria	0.91±0.09	4.66±0.24
CBU1776	Cytoplasmic	0.99±0.08	**4.99±0.27**
CBU1823	Cytoplasmic	0.90±0.25	**5.45±0.26**
CBU1825	Mitochondria	0.85±0.21	4.04±0.38
CBU1963	Cytoplasmic	0.95±0.20	4.94±0.44
CBU2052	Cytoplasmic	0.91±0.13	**5.40±0.32**
CBU2056	Cytoplasmic	0.89±0.01	4.83±0.27
CBU2059	Cytoplasmic	0.88±0.37	4.35±0.42

A Localization of ectopically expressed FLAG-tagged protein in HeLa cells (Figure 3 and Figure S4).

**Figure 4:**
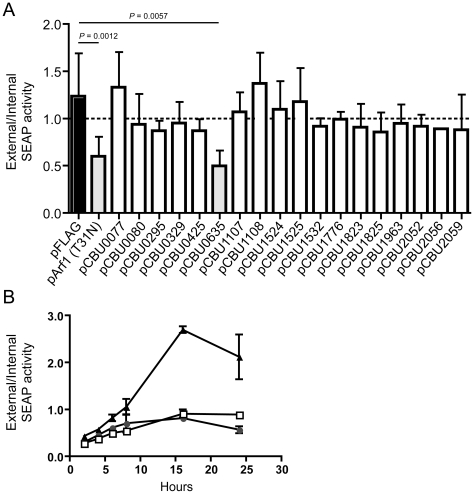
CBU0635 interferes with host protein secretion. The impact of *C. burnetii* Dot/Icm effectors on the host cell secretory pathway was examined by monitoring secretion of the SEAP protein into the culture supernatant. (A) HEK 293 cells were co-transfected with pSEAP and the plasmid encoding the indicated effector protein or the GDP-locked ARF1_T31N_ protein (pArf1-T31N) or empty vector (pFLAG). External and internal SEAP activity was measured. The grey bars show that there was a significant decrease in the external/internal ratio of SEAP activity observed upon ectopic expression of ARF1_T31N_ (P = 0.0012) or CBU0635 (P = 0.0057) in comparison to HEK 293 cells transfected with pFLAG alone (black bar). Expression of all other *C. burnetii* effectors did not significantly the ratio of SEAP activity (white bars). (B). SEAP activity (y-axis) was measured at the indicated times after cells were washed and new culture medium was added (x-axis) Data show SEAP ratios for cells with vector alone (pFLAG; black triangles), cells producing CBU0635 (pCBU0635; open squares) and cells producing ARF1_T31N_ (grey circles). A similar defect in SEAP secretion was observed in cells producing ARF1_T31N_ as in cells producing CBU0635.

B External/Internal SEAP activity (Figure 4).

**Figure 5:**
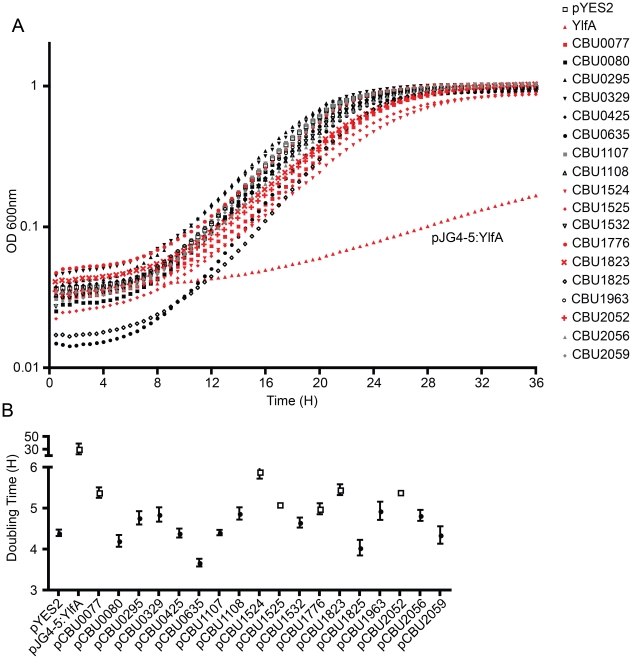
Several *C. burnetii* effectors slow yeast replication. (A) Yeast strains were grown in YNB supplemented with 2% galactose to induce the expression of the indicated effector proteins listed in the legend on the right. The plots show yeast replication as determined by measuring the optical density of the culture at 600 nm (OD 600 nm, y-axis) every 30 min (x-axis) for a period of 36 h. Growth of *S. cerevisiae* expressing *C. burnetii* effectors were compared to controls that included *S. cerevisiae* containing the vector control (pYES2; open squares) and *S. cerevisiae* producing the *L. pneumophila* effector YlfA (pJG4-5:YlfA; red triangles). Effectors that resulted in a delay in the doubling rate of *S. cerevisiae* are highlighted with red symbols. (B) The doubling time of *S. cerevisiae* producing the indicated effector proteins was calculated during the exponential phase of growth from the growth curves shown in panel A. Effectors that resulted in a significant increase in doubling time compared to *S. cerevisiae* pYES2 are highlighted in open boxes (*P*<0.01). Data represent the mean doubling time ± SD determined from at least 3 independent growth curves.

C Doubling time in hours during logarithmic phase (Figure 5).

Values in **bold** represent results significantly different to control experiments (*P*<0.01).

### CBU0635 overproduction interferes with the host secretory pathway

Several *L. pneumophila* Dot/Icm effectors have been shown to disrupt vesicle trafficking in mammalian cells by affecting the dynamics of membrane transport in the early secretory pathway [20], [21], [23], [24]. To investigate if any of the identified *C. burnetii* proteins have effector functions that interfere with the secretory pathway in mammalian cells, transport of secreted alkaline phosphatase (SEAP) was investigated in HEK 293 cells ectopically expressing the 3×FLAG-tagged effector proteins. The amount of SEAP secreted by cells into the culture medium was compared to the amount of SEAP that remained intracellular after an 8 h incubation period (Table 1 and Figure 4A). The negative control was HEK 293 cells transfected with pFLAG alone, showing an external/internal SEAP activity ratio of 1.23±0.46. The positive control was HEK 293 cells transfected with a plasmid with the GDP-locked allele ARF1_T31N_, showing a SEAP activity ratio of 0.60±0.21. Expression of the *C. burnetii* Dot/Icm effector CBU0635 was found to interfere with the secretory pathway based on an external/internal SEAP activity ratio of 0.49±0.17. Consistent with CBU0635 being the only effector that showed a pattern of localization near the Golgi apparatus when ectopically expressed in eukaryotic cells (Figure 3B), it was the only effector found to interfere with the host secretory pathway. Time course measurements determined that CBU0635-expressing cells had a defect in the kinetics of SEAP secretion that was similar to cells producing ARF1_T31N_ (Figure 4B). Thus, CBU0635 has an effector activity that when overproduced disrupts the mammalian secretory pathway.

### Several *C. burnetii* Dot/Icm effectors slow yeast replication when overproduced

Several studies have demonstrated that expression of *L. pneumophila* effector proteins in *Saccharomyces cerevisiae* can interfere with the rate of yeast replication [37]–[39]. The *L. pneumophila* effector RalF interferes with yeast replication by virtue of it being able to function as a guanine nucleotide exchange factor for ARF [37]. Inhibition of yeast replication was the basis for identification of the *L. pneumophila* Dot/Icm effectors YlfA and YlfB [37]. To test whether the *C. burnetii* Dot/Icm effectors had similar activities, these proteins were produced in *S. cerevisiae* and growth was monitored in liquid YNB supplemented with 2% galactose to induce effector expression. None of the NM Dot/Icm effectors conferred a yeast growth phenotype equivalent to that seen with expression of YlfA (Figure 5A). To determine yeast growth rates, growth curves were performed in duplicate wells in at least three independent experiments. The doubling time of each yeast strain was calculated during the exponential phase of growth between 14 h and 18 h (Figure 5B and Table 1). The negative control strain consisting of *S. cerevisiae* pYES2 had a doubling time of 4.4±0.18 h. Expression of YlfA increased the doubling time to 30.94±18.74 h, *P* = 0.013. Six *C. burnetii* effectors significantly altered the growth rate of *S. cerevisiae*; CBU0077 5.39±0.29 h (*P* = 1.9×10^−4^), CBU1524 5.90±0.39 h (*P* = 5.0×10^−5^), CBU1525 5.10±0.16 h (*P* = 5.2×10^−4^), CBU1776 4.99±0.27 h (*P* = 0.0054), CBU1823 5.46±0.29 h (*P* = 1.7×10^−4^) and CBU2052 5.40±0.32 h (*P* = 5.5×10^−4^). Thus, expression of these six *C. burnetii* proteins moderately slows yeast replication.

### The Dot/Icm secretion system is essential for *C. burnetii* intracellular replication

**Figure 6:**
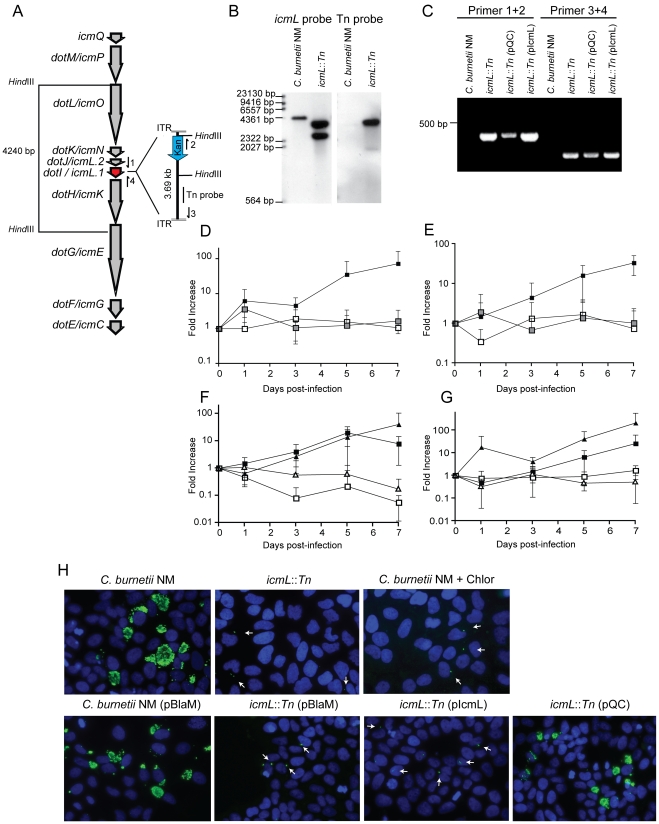
The *C. burnetii* Dot/Icm system is essential for intracellular replication. (A) A clone of *C. burnetii* with a 3.69 kb transposon inserted in the *icmL* gene (*dotI*/*icmL.1*) was isolated after enrichment on medium containing kanamycin. The schematic shows the predicted location and orientation of the transposon in *icmL* as determined by sequence analysis. The location of probes and restriction sites used to validate the site of transposon insertion by PCR and Southern hybridization are indicated. (B) As indicated, genomic DNA digested with *Hind*III isolated from the *C. burnetii* NM strain and the isogenic *icmL*::Tn mutant was analyzed by Southern hybridization using a probe derived from the *icmL* gene and a probe derived from the transposon. As predicted from the schematic in panel A, the *icmL* probe identified a single band of 4.2 kb in the NM strain, and two bands of 3.3 kb and 2.5 kb in the *icmL*::Tn mutant because of a new *Hind*III site introduced into the *icmL* locus by the transposon. When a probe homologous to the transposon was used (Tn probe), a single band was identified in the *icmL*::Tn mutant, indicating that this strain has a single insertion of the transposon in the chromosome. (C) PCR amplification of genomic DNA from NM and the *icmL*::Tn mutant using primer sets shown in panel A confirm the predicted location and orientation of the transposon insertion in *icmL*, and that the transposon is retained by the *icmL*::Tn strain after introduction of a plasmid encoding *icmL* (pIcmL) or a plasmid encoding the entire operon harboring *icmL* (pQC). (D,E) The ability of the *icmL*::Tn mutant to replicate in HeLa (D) and Vero (E) cells was determined by measuring genomic equivalents (y-axis) at the indicated times after infection (x-axis). There was a 50 to 100-fold increase over 7 days in *C. burnetii* NM genomic units (black squares). No significant increase in genomic units was detected for the *icmL*::Tn mutant (white squares) or *C. burnetii* NM treated with 10 µg/ml chloramphenicol (gray squares). (F,G) The ability of the *icmL*::Tn mutant to replicate in HeLa (F) and Vero (G) cells was determined by measuring genomic equivalents (y-axis) at the indicated times after infection (x-axis). Replication was observed for *C. burnetii* NM containing pBlaM (black squares) and the *icmL*::Tn mutant containing the plasmid pQC (black triangles). The *icmL*::Tn mutant containing the vector pBlaM (white squares) or for the *icmL*::Tn mutant containing pIcmL (white triangles). Graphs represent the mean ± SD of at least three independent experiments. (H) Fluorescence micrographs were acquired after infection of HeLa cells for 5 days by the strains of *C. burnetii* indicated. An anti-*Coxiella* antibody (green) was used to visualize intracellular bacteria and the nucleus of the host cell was visualized by DAPI staining (blue). Replicating bacteria in large vacuoles were observed for cells infected with *C. burnetii* NM, *C. burnetii* NM containing pBlaM and the *icmL*::Tn mutant containing pQC. Only individual bacteria were observed inside the host cells infected with the *icmL*::Tn mutant, *C. burnetii* NM in medium with chloramphenicol, *icmL*::Tn containing pBlaM and *icmL*::Tn containing pIcmL (indicated with arrows). These are representative images from at least three independent experiments.

To determine the cumulative role effector proteins may play during infection of host cells by *C. burnetii*, a genetic screen was initiated to identify *C. burnetii* having a gene essential for Dot/Icm transporter function disrupted. Towards this end a *mariner*-based *Himar1 C9* transposase system was used to generate random insertions in the chromosome of *C. burnetii* NM [40]. Kanamycin-resistant transposon mutants of *C. burnetii* were selected on defined ACCM medium and then screened by PCR for insertions into the *dot/icm* locus. A mutant was isolated with the transposon inserted at base 1569472 in the chromosome, disrupting the *icmL* gene (also called *icmL.1* and *dotI*) predicted to be essential for function of the Dot/Icm system (Figure 6A). Southern blot analysis and PCR amplification of the insertion site was used to confirm the site of transposon integration and to determine that there was a single copy of the transposon in this mutant (Figure 6B and C).

To determine whether inactivation of the Dot/Icm system would influence the ability of *C. burnetii* to replicate within eukaryotic host cells, both HeLa and Vero cells were infected at a multiplicity of infection (MOI) of 50 with either the parental strain *C. burnetii* NM phase II or the isogenic *icmL*::Tn mutant and bacterial replication was assayed over a period of seven days (Figure 6D and E). No replication of the *icmL*::Tn mutant was observed in either cell line. The isogenic *C. burnetii* NM phase II strain with a functional Dot/Icm system demonstrated a 100-fold increase in genome equivalents over the same period. Immunofluorescence microscopy of infected HeLa cells stained with an anti-*Coxiella* antibody revealed the formation of large CCVs containing replicating *C. burnetii* NM, and a complete absence of large vacuoles containing replicating bacteria for the isogenic *icmL*::Tn mutant (Figure 6H). The intracellular replication defect observed for the isogenic *icmL*::Tn mutant was similar to that observed for *C. burnetii* NM phase II cultured with host cells in medium containing chloramphenicol (Figure 6D, E and H). Importantly, the *icmL*::Tn mutant grew at an equivalent rate to the control strain of *C. burnetii* in the defined medium ACCM (Figure S6), and the intracellular replication defect was not a consequence of reduced entry, as the *icmL*::Tn mutant had an equivalent capacity to enter HeLa cells as the isogenic control strain (Figure S6). Furthermore, viable *icmL*::Tn mutant bacteria were recovered from HeLa cells at 24 h, 72 h and 120 h post-infection (Figure S6), suggesting that the Dot/Icm apparatus is not essential for survival within HeLa cells.

Complementation studies validated that the defect in replication of the *icmL*::Tn mutant resulted from a loss of Dot/Icm system function. The *icmL* gene is centrally located in a putative operon that begins with the *icmQ* gene and terminates after the *icmC* gene (Figure 6A), which would predict that polar effects resulting from the insertion of the transposon in the *icmL* gene would also limit the production of the downstream *icmKEGC* gene products. For this reason, complementation studies were conducted using two different plasmids. The plasmid pIcmL contained only the *icmL* gene, whereas, the plasmid pQC contained the entire operon from *icmQ* to *icmC*. The ability of these plasmids to complement the *icmL*::Tn mutation was determined by assaying intracellular replication of the resulting *C. burnetii* strains containing these plasmids in HeLa and Vero cells compared to a positive control strain, which was *C. burnetii* NM containing a plasmid encoding the ß-lactamase gene (pBlaM). Intracellular replication of the *icmL*::Tn mutant was fully restored by the plasmid pQC as indicated by the increase in genome equivalents over time (Figure 6F, G) and the appearance of vacuoles containing replicating bacteria at day 5 post-infection (Figure 6H). Thus, the intracellular growth defect observed for the *icmL*::Tn mutant is linked genetically to the region of the transposon insertion and is caused by inactivation of the Dot/Icm system. As predicted, the plasmid pIcmL did not complement the *icmL*::Tn mutation, consistent with the tranposon insertion having a polar effect on production of the downstream *icm* gene products. These studies demonstrate that the Dot/Icm system and the cumulative activities of the translocated effectors are essential for establishment of a vacuole that supports *C. burnetii* replication in mammalian host cells.

### Demonstration of Dot/Icm-dependent protein translocation in *C. burnetii*

**Figure 7:**
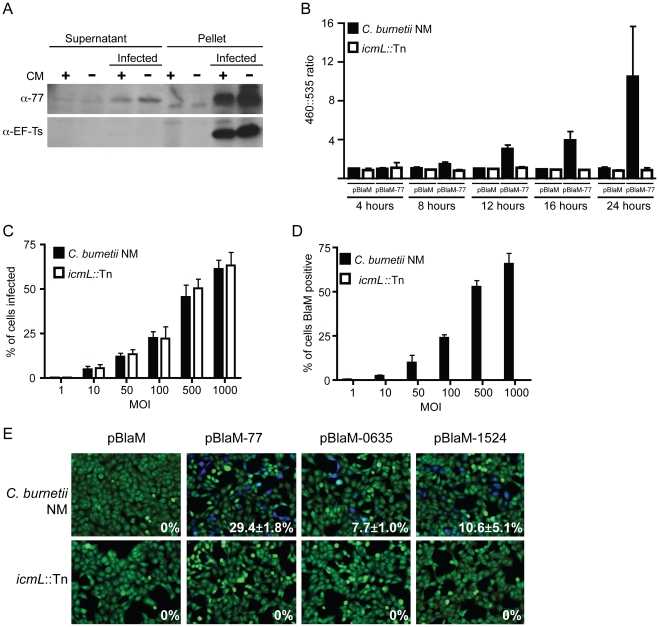
The *C. burnetii* Dot/Icm secretion system is required for effector translocation. (A) HeLa cells either infected with *C. burnetii* NM or uninfected were incubated with or without chloramphenicol (CM) and cellular lysates were separated into supernatant and pellet fractions. Immunoblot analysis was used to detect CBU0077 (top panel; α-77) and EF-Ts (bottom panel; α-EF-Ts). The CBU0077 protein was detected in both the supernatant fraction containing proteins secreted during infection and in the pellet fraction containing intact bacteria. The cell associated EF-Ts protein was detected only in the pellet fraction containing intact bacterial cells. The addition of chloramphenicol resulted in a slight drop in levels of CBU0077 detected in the supernatant, suggesting the secreted protein has a relatively long half-life. Similar results were obtained from three independent experiments. (B) *C. burnetii* NM (black bars) and the *icmL*::Tn mutant (white bars) containing either pBlaM or pBlaM-77 were used to infect HeLa cells for the times indicated (x-axis). Translocation of the BlaM and BlaM-CBU0077 fusion protein by the respective strains was determined by measuring the change in the 460 nm/535 nm fluorescence emission ratio resulting from cleavage of the CCF4-AM substrate (y-axis). Results represent the mean ± SD obtained from triplicate samples. (C, D) HeLa cells were infected in parallel with *C. burnetii* NM producing BlaM-CBU0077 or the *icmL*::Tn strain producing BlaM-CBU0077 at the multiplicities of infection (MOI) indicated on the x-axis. Fluorescence microscopy was used to determine the percent of cells infected by the two strains (C) and the number of cells that stained positive for BlaM translocation (D) Results represent the mean ± SD obtained from triplicate samples. (E) Fluorescence micrographs of HeLa cells that were infected for 24 h with either *C. burnetii* NM or the *icmL*::Tn strains producing either BlaM alone or BlaM fusions to CBU0077 (pBlaM-77), CBU0635 (pBlaM-0635) or CBU1524 (pBlaM-1524). Emission at 535 nm (green) identifies the CCF4-AM-loaded cells and emission at 460 nm (blue) indicates cleavage of CCF4-AM in the cytosol of the infected cell resulting from translocation of the BlaM fusion protein. The percent of cells that were BlaM positive (blue) was determined for each infection condition and the mean ± SD from three independent experiments is displayed in the bottom right corner of each representative micrograph.

To validate that the effectors identified in this screen are delivered into host cells during infection by *C. burnetii*, a polyclonal antibody specific for the conserved *C. burnetii* Dot/Icm effector CBU0077 was generated (Figure 7A). HeLa cells persistently infected with *C. burnetii* were separated into a soluble fraction that contained host proteins and bacterial proteins secreted or translocated during infection, and a pellet fraction containing *C. burnetii* proteins that remain associated with intact bacterial cells and insoluble host debris. Immunoblot analysis revealed CBU0077 in the fraction of secreted/translocated bacterial proteins, whereas, the *C. burnetii* translation elongation factor EF-Ts, which is not a secreted/translocated protein, was detected only in the pellet fraction. These data indicate CBU0077 is produced and translocated out of the bacterial cell during infection. When *de novo* synthesis of bacterial proteins in the infected cells was prevented by treatment with chloramphenicol for 16 h prior to fractionation, a slight decrease in the amount of CBU0077 was found for the secreted fraction and the pellet fraction, suggesting that CBU0077 has a relatively long half-life in both bacterial and host cell compartments (Figure 7A).

To independently address whether CBU0077 is translocated during infection, a ß-lactamase-based translocation assay was used. In this assay, a derivative of ß-lactamase (BlaM) that lacks a signal sequence is fused to the amino terminus of a potential Dot/Icm effector. The delivery of a BlaM hybrid into the host cytosol is monitored using the substrate CCF4-AM. When BlaM fusion proteins are delivered into the host cell cytosol the CCF4-AM molecule is cleaved, resulting in a shift in fluorescence emission from 535 nm (Green) to 460 nm (Blue) when excited at 415 nm. Plasmids encoding *blaM* alone and the *blaM-CBU0077* fusion were introduced into *C. burnetii* NM phase II and the isogenic *icmL*::Tn mutant. Production of the expected BlaM proteins was confirmed by immunoblot analysis (Figure S7). These *C. burnetii* strains producing BlaM proteins were used to infect HeLa cells, and cleavage of the CCF4-AM substrate was measured at 4 h, 8 h, 12 h, 16 h and 24 h post infection (Figure 7B). Translocation of BlaM and BlaM-CBU0077 was undetectable at 4 h post infection in all strains examined. At 8 h post infection, translocation of BlaM-CBU0077 was detected by *C. burnetii* NM phase II, with a 460 nm/535 nm fluorescence emission ratio relative to uninfected cells of 1.58±0.28 compared to 1.06±0.22 for the strain expressing BlaM alone. This signal continued to increase at 12 h (3.03±0.43), at 16 h (3.90±0.95) and at 24 h (9.17±4.96). Translocation of the BlaM protein alone by *C. burnetii* was not detected for any strain at any time point, confirming that delivery of BlaM into the host cytosol required fusion to the type IV effector CBU0077. Most importantly, translocation of BlaM-CBU0077 was not detected using the *icmL*::Tn mutant, demonstrating that the Dot/Icm apparatus is essential for delivery of this effector into the host cytosol.

Single cell assays were conducted to compare the efficiency of *C. burnetii* entry with the efficiency of effector translocation. Host cells were infected at different multiplicities with isogenic *C. burnetii* NM strains producing BlaM-CBU0077, and fluorescence microscopy was used to measure the efficiencies of infection and effector protein translocation. As the multiplicity of infection increased there was a dose-dependent increase in the number of cells that were infected by *C. burnetii* (Figure 7C). There was no difference observed in the infection efficiencies of the control *C. burnetii* NM strain compared to the isogenic *icmL*::Tn mutant. A dose-dependent increase in the number of cells receiving the BlaM-CBU0077 protein that closely mirrored the uptake efficiency was observed after infection with the control *C. burnetii* NM strain (Figure 7D), as determined by counting cells with a positive emission signal at 460 nm resulting from cleavage of the CCF4-AM substrate by the translocated fusion protein. No translocation of BlaM-CBU0077 was observed after infection of cells with the *icmL*::Tn mutant, even though uptake was similar to that observed for the isogenic *C. burnetii* NM control. These data indicate that most of the cells that are infected by *C. burnetii* NM are subject to Dot/Icm-dependent translocation of CBU0077.

The delivery of BlaM fusions to CBU0635 and CBU1524 was also assessed to validate that other effectors identified in this study were translocated into host cells during infection by *C. burnetii* by a Dot/Icm-dependent mechanism. BlaM-CBU0635 and BlaM-CBU1524 were produced in roughly equal amounts in *C. burnetii* NM and the isogenic *icmL*::Tn mutant (Figure S7). HeLa cells were scored for translocation of the BlaM fusion proteins after a 24 h infection period. Translocation-positive cells were detected after infection of host cells with *C. burnetii* NM producing BlaM-CBU0077, BlaM-CBU0635 and BlaM-CBU1524, however, there were no translocation-positive cells detected after host cells infection with the isogenic *icmL*::Tn mutant producing these fusion proteins (Figure 7E). Thus, effectors identified using the *L. pneumophila* system are engaged and translocated into host cells during *C. burnetii* infection by a Dot/Icm-dependent mechanism.

## Discussion

In this study a random screen was conducted to identify novel effectors produced by *C. burnetii*. Because type IV secretion systems recognize translocation signals at the carboxyl terminus of effectors, a strategy was employed in which a Cya reporter that lacks a translocation signal was used to monitor the presence of coding determinants fused at the carboxyl terminus of the protein that enabled Cya to be engaged and delivered into host cells by the Dot/Icm system. This approach was successful at identifying *C. burnetii* proteins delivered into host cells by the Dot/Icm system, demonstrating the feasibility in using type IV translocation signal screening as a means to identify effectors.

Four of the effector proteins identified here (CBU1524, CBU1823, CBU1825 and CBU2052) were reported recently as Dot/Icm substrates in an independent study that was based on identifying effectors based on sequence motifs and protein interaction properties [41], however, the majority of the effectors identified using the type IV secretion signal screen were not uncovered using these predictions. This highlights the benefit of using functional screens for type IV secretion signals as a means to identify new effectors. It was also reported recently that the plasmid QpH1 in *C. burnetii* encodes several Dot/Icm effectors [42]. Interestingly, none of the effectors identified here using the type IV secretion signal screen were plasmid encoded. This may reflect a low abundance of QpH1 in the genomic DNA preparation used to make the Cya fusion protein library. Other reasons for effectors not being identified in this screen for type IV secretion signals include a lack of suitable *SauIIIA* sites that would be needed for in an in-frame fusion to Cya, and the potential to miss effectors translocated at low levels because the screening approach relied on a strong signal being generated by a single clone in a mixed pool. These limitations should be considered in any future screens for type IV secretion signals, and with slight modifications to the DNA fragmentation method and screening protocol, minor improvements should enable more complete coverage of the genome.

An analysis was performed in which the effectors found in the NM strain were compared to genes from other sequenced strains of *C. burnetii*. As was observed for members of the Ank family of effector proteins [25], a significant degree of heterogeneity was revealed among the Dot/Icm effectors identified in this study. Interestingly, what appears to be effector pseudogenization is prevalent in strains of *C. burnetii* capable of causing acute and chronic Q fever in humans. The evolutionary and pathogenic significance toward Dot/Icm effector pseudogenization remains unclear. Dugway possesses the largest genome and fewest pseudogenes of the sequenced *C. burnetii* isolates [31]. This implies that a large repertoire of functional effectors could be beneficial, as it may enable *C. burnetii* to persist and be transmitted by many different animals in nature without causing disease to their hosts. The loss of effectors appears to correlate with a change in the relationship between *C. burnetii* and mammalian hosts from commensalism to parasitism, which would indicate that a balanced repertoire of effectors is important in preventing responses that would result in host pathology.

Further investigation is required to determine whether the truncated NM Dot/Icm effectors identified here are non-functional or whether these genes still produce functional effectors that mediate the same or distinct activities compared to the larger proteins predicted to be produced by alleles found in other strains of *C. burnetii*. Because effectors are modular proteins that can have multiple enzymatic domains with different effector functions, it remains possible that some of the mutations resulting in truncation of a NM protein occur through positive selection, where the truncated effector has evolved in such a way that it retains a beneficial enzymatic activity and discards an activity that is no longer being selected for in the infected host. CBU0080 and the homologue CbuK1976 may provide such an example. Ectopically expressed CbuK1976 has an affinity for the host nucleus, whereas, the truncated NM protein CBU0080, which lacks a helix loop helix motif found in CbuK1976, is retained in the cytoplasm. Conceivably, CBU0080 could be a functional effector where elimination of the helix loop helix motif is the result of positive selection as it may enhance an activity associated with the C-terminal region of the protein. The effectors CBU1107 and CBU1108 provide another intriguing comparison. CBU1107 and CBU1108 have independent determinants that confer Dot/Icm-dependent translocation. In Dugway, genes encoding these two effectors are fused to generate the large protein CBUD1210, which would represent a hybrid effector. It is possible that a mutation in NM resulting in two gene products, CBU1107 and CBU1108, was positively selected because it allows more precise regulation of two effector functions compared to the hybrid protein CBUD1210. Alternatively, CBUD1210 could represent fusion of the two genes encoding CBU1107 and CBU1108 and this fusion was positively selected because it links two effectors that may act in concert. Although these examples remain highly speculative, it illustrates how studies on *C. burnetii* effector evolution could provide novel insight into how the repertoire of Dot/Icm effectors is being shaped by host interactions.

Several approaches were used to investigate whether the identified effector proteins had activities that would perturb eukaryotic cell functions (summarized in Table 1). *C. burnetii* Dot/Icm effector proteins are likely to modulate a broad array of eukaryotic cellular processes. This is supported by the diversity observed in the localization of these effectors produced in mammalian host cells. Although several effectors were found diffusely in the cytosol of the mammalian cell, there were clear examples of specific localization of effectors to the nucleus, mitochondria, lysosomes and a perinuclear region near the Golgi apparatus. Consistent with its localization near the Golgi apparatus, the effector CBU0635 disrupted membrane transport in the host secretory pathway as determined by a defect in SEAP secretion. The localization of CBU0077 with lysosomes was also intriguing, as the CCV is an organelle derived from lysosomes. The observation that CBU0077 is highly conserved in all sequenced strains of *C. burnetii* makes this protein an attractive candidate for being directly involved in processes important for CCV biogenesis. Isolation of a *C. burnetii* mutant deficient in CBU0077 may provide some clues as to the role this effector plays in modulating host processes during infection.

Six of the *C. burnetii* effectors were found to moderately affect the replication of yeast when overproduced. These differences were minor compared to what is observed when many of the *L. pneumophila* effectors are produced in *S. cerevisiae*, where replication and viability are affected so severely that the yeast are unable to form single colonies on agar plates. Because of the severity of these defects, several *L. pneumophila* Dot/Icm effector proteins were identified based on their capacity to interfere with yeast viability [37]. Furthermore, examination of the impact of a large cohort of *L. pneumophila* Dot/Icm effectors showed that in excess of 60% of these proteins significantly decrease yeast growth and/or viability [38]. The observation that none of the *C. burnetii* effectors had this dramatic effect on yeast viability could relate to the different roles the Dot/Icm system is likely to play during infection by these two pathogens. For *L. pneumophila*, the Dot/Icm system has evolved to modulate cellular functions in evolutionarily diverse protozoan hosts and to create a vacuole that is derived from the endoplasmic reticulum. Many of the targets that have been identified for *L. pneumophila* effectors are proteins that are highly conserved throughout the eukaryotic kingdom. By contrast, *C. burnetii* infects mammals and has reprogrammed its effector repertoire to survive and replicate inside mammalian host cells. As such, there are likely to be effectors that have evolved to manipulate targets specific to mammalian cells and may not recognize targets in more primitive eukaryotic cells, such as yeast. An example of this is the effector AnkG that blocks apoptosis by targeting the host protein p32, which is found in mammals but not in yeast or protozoa [26].

Studies on the effector CBU0077 showed that this protein was produced during infection. Both the endogenous CBU0077 protein and the BlaM-CBU0077 fusion protein were delivered into host cells. Because the BlaM detection system is based on delivery of a robust enzyme into the host cytosol, the assay is sensitive and can detect translocation of small amounts of a fusion protein. Detectible levels of effector translocation were first detected at 8 h post infection. The inability to detect translocation of BlaM-CBU0077 at 4 h suggests that the *C. burnetii* Dot/Icm system is not fully functional during the early stages of host cell infection. Bacterial proton motive force is involved in the translocation of effectors by the *L. pneumophila* Dot/Icm system [43], which implies that bacterial metabolism is important for the functioning of this apparatus. It has been shown that infectious forms of *C. burnetii* that are environmentally resistant do not become metabolically active until they gain access to a low pH environment [3], [44], [45]. Thus, it is possible that the Dot/Icm system is not functioning in the early events in vacuole biogenesis and only becomes active once the *C. burnetii* have been transported through the endocytic pathway of the host cell to an acidified lysosomal compartment. There are several logical reasons why the Dot/Icm system may not be needed by *C. burnetii* during these early stages of infection. Because fusion of phagosomes with lysosomes represents a default membrane transport pathway that most bacteria follow after uptake, the Dot/Icm system should not be needed to promote delivery of the bacteria to this preferred location. Additionally, it is now well established that mammalian cells have several innate immune sensors that can detect the activities of the *L. pneumophila* Dot/Icm system during the early stages of infection [46], [47]. Thus, not having the Dot/icm system functioning during these early infection events may help delay or prevent innate immune detection of *C. burnetii*.

Recent advances in axenic cultivation of *C. burnetii* [48] and newly developed genetic tools developed to study this organism [40] were used to isolate a transposon insertion mutant that lacked a functional Dot/Icm apparatus. As expected, the BlaM-CBU0077, BlaM-CBU0635 and BlaM-CBU1524 proteins were not delivered into host cells by the *icmL*::Tn mutant, providing additional evidence that translocation of these effectors by *C. burnetii* requires a functional Dot/Icm system. Similar to what was shown initially for *L. pneumophila dot* and *icm* mutants [12], [13], the ability of the *C. burnetii icmL*::Tn mutant to grow on synthetic medium as efficiently as the isogenic parental strain demonstrates conclusively that the Dot/Icm system is not essential for replication extracellularly. Most importantly, when the *icmL*::Tn was used to infect mammalian cells, there was a severe defect in the ability of this mutant to replicate intracellularly that was linked genetically to the inactivation of the Dot/Icm system by insertion of the transposon.

These data demonstrate that the Dot/Icm system plays an essential role in the process of intracellular infection of mammalian host cells, which means that the collective activities of the effector proteins delivered into cells by the Dot/Icm system are needed for the successful establishment and maintenance of the specialized vacuole in which this pathogen resides. Thus, determining the biochemical activities of the novel *C. burnetii* effectors identified in this study should help elucidate the mechanisms by which this intracellular pathogen can persist and grow inside mammalian host cells.

## Methods

### Reagents

Unless otherwise noted, chemicals were purchased from Sigma (St. Louis, MO). Restriction enzymes and molecular cloning enzymes were purchased from New England Biolabs (NEB; Ipswich, MA). Cell culture media and fetal bovine serum (FBS) was obtained from Invitrogen (Carlsbad, CA). Protease inhibitor cocktail and Fugene 6 transfection reagent were from Roche Applied Sciences (Indianapolis, IN).

### Host cell lines and bacterial strains

CHO cells expressing FcγRII [28], HeLa 229, Vero and HEK 293 cells were maintained in Dulbecco's Modified Eagle's Media (DMEM) supplemented with 10% heat-inactivated FBS at 37°C in 5%CO_2_ unless otherwise described.

A plaque-purified isolate of *Coxiella burnetii* phase II Nine Mile strain, a generous gift from Dr. T. Hackstadt of the Rocky Mountain Laboratories (Hamilton, MT) was propagated in eukaryotic cell lines in DMEM supplemented with 5% FBS at 37°C in 5% CO_2_ or ACCM at 37°C in 5% CO_2_ and 2.5% O_2_ as described previously [48]. When required chloramphenicol and kanamycin were used in *C. burnetii* ACCM cultures at 3 µg/ml and 275 µg/ml respectively.

*Legionella pneumophila* strains, CR39 and CR58 ( Δ*dotA*), which are isogenic derivatives of the serogroup 1 strain LP01 [13], [49], were grown on either charcoal-yeast extract (CYE) plates or in ACES-buffered yeast extract (AYE) broth containing chloramphenicol (6.25 µg/ml) as previously described [50]. *E. coli* strains were cultivated on Luria-Bertani (LB) plates or broth supplemented with chloramphenicol (25 µg/ml), ampicillin (100 µg/ml) or kanamycin (50 µg/ml) as appropriate.

### Construction of *C. burnetii* genomic DNA library

Purified *C. burnetii* were pelleted for 5 min at 8000×g in a microcentrifuge. Pellets were resuspended in 50 mM Tris-HCl, pH 8.0 containing 50 mM EDTA, pH 8.0. Lysozyme was added to a final concentration of 5 mg/ml and incubated at RT with gentle rocking. After 30 min, sodium dodecyl sulfate (SDS) and proteinase K (Roche Applied Sciences) were added to final concentrations of 1% and 10 mg/ml, respectively, and incubated for 1 h at RT with gentle rocking. Genomic DNA was extracted with phenol, phenol-chloroform, and chloroform; precipitated with ethanol; air dried; then resuspended in 10 mM Tris-HCl, pH 8.0 containing 1 mM EDTA, pH 8.0. Genomic DNA was partially digested with *Sau*3AI for 1 h at 37°C, separated by gel electrophoresis, and purified from a Low Melt agarose gel (American Bioanalytical, Natick, MA) as follows. DNA fragments between 2 kb and 4 kb were excised from the gel, placed in eppendorf tubes, and heated to 70°C for 10 min. An equal volume of prewarmed phenol (40°C) was added to the melted agarose, mixed, and separated by 5 min centrifugation at 16,000×g. The upper aqueous layer was chloroform extracted, ethanol precipitated and resuspended in 10 mM Tris-HCl, pH 8.0 containing 1 mM EDTA, pH 8.0.

Plasmid, pEC33, was generated from pM45-*ralF* [27] as follows: plasmid was digested with *Bam*HI and *Pst*I, gel purified to remove *ralF* gene insert, treated with DNA polymerase I (Klenow) fragment, and ligated to preserve the *Bam*HI site (E. Cambronne, unpublished).

*C. burnetii* genomic DNA fragments were ligated into *Bam*HI digested pEC33 resulting in a library of *C. burnetii* genes fused to the carboxyl region of the enzymatic domain of the *Bordetella pertussis* adenylate cyclase toxin. The genomic library was transformed into ElectroMax DH10B electroporation competent *E. coli* (Invitrogen). The insert efficiency was estimated to be 92% by restriction enzyme digest of randomly selected clones. Following amplification in *E. coli*, the library DNA was purified by cesium chloride density fractionation and subsequently used to transform *L. pneumophila*.

### Screening of genomic DNA library

To screen the *C. burnetii* genomic DNA library for translocation into mammalian host cells using the Cya fusion strategy [51], *L. pneumophila* strain CR39 was transformed with the library of *C. burnetii* genes fused to *cya* by electroporation and the library of transformants was expanded on CYE plates. The library was distributed into pools containing approximately 50 transformants in each, and the pools were expanded by growing the bacteria on 24 well plates of CYE supplemented with chloramphenicol for 4 days at 37°C. Bacteria from each well were resuspended in AYE medium, an equal volume of freeze media (4% (w/v) peptone containing 10% (v/v) glycerol) was added, and the pools were stored in a 96-well plate format at −80°C.

Briefly, the translocation assay was performed as follows (see Figure S2). Pools of *L. pneumophila* expressing Cya-*C. burnetii* fusion proteins growing on CYE in 24 well plates were resuspended in AYE to a concentration of 1×10^9^ cfu/ml. CHO- FcγRII cells were pretreated for 1 h with an anti-*Legionella* antibody then infected with 3×10^6^ *L. pneumophila* from each pool. After 1 h of incubation at 37°C, monolayers were washed three times with ice-cold PBS and lysed in cold buffer containing 50 mM HCl and 0.1% (v/v) TX-100 for 30 min at 4°C. The lysates were boiled for 5 min and neutralized with 30 mM NaOH. Total cAMP was extracted and quantified using cAMP Biotrak Enzymeimmunoassay System (GE Healthcare, Piscataway, NJ). Pools that registered cAMP levels of 2.5 times over vector control were considered positive.

Individual colonies from pools that were scored positive in the cAMP assay were isolated and arrayed in 96 well plates containing CYE agar. Secondary screening was conducted by pooling the 12 clones in each row to identify which row contained a positive clone (Figure S2B). Rows that gave a positive cAMP signal were further analyzed by testing individual clones present in the positive rows for translocation (Figure S2C).

Plasmid DNA from individual *L. pneumophila* transformants that were identified using the above screening procedure was isolated as follows. Bacteria from a confluent region on a CYE plate were used to inoculate AYE containing chloramphenicol and grown overnight at 37°C. Bacteria were pelleted and resuspended in STET buffer (8% (w/v) sucrose, 50 mM Tris-HCl, pH 8.0, 50 mM EDTA, pH 8.0, 0.1% (v/v) Triton X-100). Fresh lysozyme (2 mg/ml) was added and incubated for 5 min at RT before boiling the samples for 1 min. Plasmid DNA was isolated from aqueous phase using Miniprep Express Matrix (Bio101, La Jolla, CA) as per manufacturer's instructions. To sequence fusion junctions, plasmid DNA was isolated from *E. coli* transformants as described above and sequenced by W.M Keck Foundation Biotechnology Resource Laboratory (Yale University).

### Plasmid construction

Full-length genes of constructs containing in-frame fusions of putative effectors were amplified by PCR with sequence specific primers containing *Bam*HI and *Pst*I sites for cloning into pEC33 (Table S3). pcDNA4/TO (Invitrogen) with a N-terminal 3×FLAG tag (pFLAG) was used to clone Dot/Icm effectors from NM, Dugway, G and K isolates through *Bam*HI and *Xho*I or *Bam*HI and *Eco*RI for CBU0295 and CBU1823 or *Bam*HI and *Xba*I for CBU2059 (Table S3). Plasmids encoding in frame fusions between the coding regions of BlaM and the effector proteins were generated by inserting the gene encoding the effector at a *SalI* site located at the 3′ end of the *blaM* gene in the plasmid pJB-CAT-BlaM [42] using the In-Fusion Advantage PCR Cloning system as described by the manufacturer (Clontech Laboratories, Palo Alto, CA). For complementation studies the *icmL* gene and the *icmQPONL2L1KEGC* operon were ligated into the plasmid pJB-CAT-BlaM after digestion of the DNA with *Pst*I and *Sal*I to remove the *blaM* gene, generating the plasmids pIcmL and pQC, respectively.

### RT-PCR analysis

Total RNA was extracted from *C. burnetii* purified from persistently infected CHO- FcγRII cells using a Trizol Max Bacterial Isolation kit (Invitrogen) as per manufacturer's instructions. RNA was treated with RNase-free DNase (Qiagen, Valencia, CA) and purified using RNeasy mini spin columns (Qiagen) as per manufacturer's instructions for RNA cleanup. First strand cDNA was synthesized from 5 µg total RNA using gene specific RT primers (Table S3) with the Superscript III First Strand Synthesis System for RT-PCR (Invitrogen). PCR reactions were performed from cDNA prepared with or without Superscript III reverse transcriptase using gene specific primers and Elongase enzyme mix (Invitrogen).

### Fluorescence microscopy

To examine the localization of ectopically produced FLAG-tagged proteins, HeLa 229 or CHO- FcγRII cells were plated on 12 mm glass coverslips in 24-well plates at a density of 3×10^4^ cells/well. Cells were transfected with plasmid DNA using Fugene 6 (Roche) or Lipofectamine 2000 (Invitrogen). Eighteen hours post-transfection cells were fixed using 4% paraformaldehyde and permeablized with Triton X-100. Samples were incubated with primary antibodies in 2% BSA at the following concentrations; anti-FLAG (Sigma) 1∶300, anti-LAMP1 H4A3 (Developmental Studies Hybridoma Bank) 1∶50, anti-TOM22 (Sigma) 1∶250, anti-GM130 (BD) 1∶250, anti-*C. burnetii* 1∶10000. Secondary antibodies, Alexa Fluor 488 and 546 (Invitrogen) were used at 1∶2000 in 2% BSA. Bacterial and host cell DNA was labeled using 0.1 µg/ml 4,6-diamidino-2-phenylindole (DAPI). Coverslips were mounted on slides using ProLong Gold (Invitrogen). Images were acquired with a Zeiss LSM510 microscope using a 100×/1.4 numerical aperture objective. Images were exported as TIFF files and labeled with Adobe Illustrator. Similar results were obtained in at least three independent experiments.

### CBU0077 translocation assay

Uninfected or *C. burnetii*-infected HeLa cells were incubated with chloramphenicol (25 µg/ml) or DMSO for 16 h at 37°C in 5%CO_2_. Cells were washed with Hanks buffered salt solution (HBSS, Invitrogen) containing 1× protease inhibitor cocktail (PI) then lysed with ice-cold HBSS containing PI and 0.1% Triton X-100. The cells were centrifuged for 10 min at 16,000g at 4°C to separate the soluble fraction containing secreted and translocated bacterial proteins from the insoluble fraction, which contained intact bacteria. The soluble fraction was clarified using a 0.22 µm pore-size filter (Millipore, Bedford, MA) and proteins were concentrated by trichloroacetic acid precipitation. Samples from both the soluble and insoluble fractions were separated by SDS-PAGE, transferred to PVDF (Millipore) and probed with anti-CBU0077 or anti-EF-Ts [52] as a bacterial lysis control. Similar results were obtained from three independent experiments.

### Yeast growth assay

Genes encoding Dot/Icm effectors were amplified from NM gDNA by PCR with sequence specific primers containing a *Bam*HI site, Kozak sequence and ATG, if applicable, at the 5′ end, and an *Xba*I or *Sph*I at the 3′end (Table S3). The PCR products were cloned into pYES2 (Invitrogen), and introduced in competent *S.cerevisiae*. Overnight liquid YNB-glucose cultures were pelleted and washed once with YNB before being resuspended to an OD 600 nm of approximately 0.03 in YNB-galactose. 200 µl of this suspension was distributed into duplicate wells of a 96 well tray and incubated in a Tecan M1000 plate reader at 30°C. OD 600 nm readings were taken every 30 minutes, following a 30 second agitation, for the duration of the time course. Growth curves were created from at least three independent experiments.

### SEAP assay

HEK 293 cells in 24 well plates were co-transfected with 0.2 µg pSEAP and 0.3 µg of a pFLAG derivative encoding the indicated Dot/Icm effector or control protein. Approximately 16 h post-transfection the cells were washed with fresh medium, and a sample was collected to calculate the background SEAP activity in the culture supernatant. Following an 8 h incubation period, the culture medium was collected to assay SEAP levels secreted by the cells, and an equivalent volume of buffer containing 0.2% Triton X-100 was applied to the cells to measure the intracellular alkaline phosphatase activity. Alkaline phosphatase activity was measured in 96-well format using the Phospha-Light System (Applied Biosystems). Ratios of extracellular to intracellular SEAP activity were calculated from at least three independent experiments.

### Genetic manipulation of *C. burnetii*

Plasmid DNA, for introduction into *C. burnetii*, was purified using the GeneElute HP Endotoxin Free Plasmid Maxiprep Kit (Sigma) and concentrated with an Amicon Ultra Centrifugal Filter (Millipore). This DNA was introduced into axenically grown *C. burnetii* by electroporation. Approximately 5×10^9^ bacteria were washed twice and resuspended in ice-cold 10% glycerol. Plasmid DNA was added (10 µg) and the bacteria were electroporated at 18 kV, 500Ω, 25 µF. Electroporated *C. burnetii* were resuspended in 1 ml RPMI and 200 µl of this was used to seed a 20 ml ACCM culture. After a 24 h recovery, the appropriate antibiotics were added. Cultures were passaged after 7 days. Following 14 days of selection in liquid ACCM the bacteria were grown on ACCM agarose plates to isolate individual transformants.

### Southern hybridization

PCR probes for *icmL* and the transposon were amplified, with the incorporation of digoxigenin-dUTP, using the primer pairs *icmL.1*F/*icmL.1*R and TnF/TnR respectively (Table S3). gDNA was digested with *Hind*III, separated by agarose gel electrophoresis and transferred to positively charged nylon membrane. Hybridization was performed overnight at 60°C and DIG detection was performed according to the manufacturers' guidelines (Roche).

### BlaM translocation assay

CBU0077, CBU0635 and CBU1524 were introduced into the *Sal*I site of pJB-CAT-BlaM to create a BlaM fusion constructs under the control of the CBU1169 promoter. These constructs were electroporated into *C. burnetii* and individual clones isolated after two passages with chloramphenicol selection. BlaM expression was confirmed by western blot with anti-BlaM (1∶1000, QED Bioscience Inc, San Diego, CA). Approximately 2×10^4^ HeLa cells were seeded in black clear bottom 96 well trays (Corning Incorporated, Corning NY). The next day monolayers were infected with *C. burnetii* at the appropriate MOI and the infection allowed to proceed until the desired time point. Cells were loaded with the fluorescent substrate CCF4/AM, using the LiveBLAzer-FRET B/G Loading Kit (Invitrogen) with 15 mM probenecid, in the dark for 2 h at room temperature. Fluorescence, using an excitation of 415 nm and emission at 460 nm and 535 nm, was quantified using the Tecan M1000 plate reader. Following background subtraction the ratio of 460 nm to 535 nm (blue∶green) was calculated and expressed relative to uninfected cells. For single cell assays, the infection and stain procedure was similar. Cells were visualized by fluorescence microscopy using an inverted Nikon Eclipse TE-2000 S microscope and a 20× objective. A total of 400 cells were counted in three independent wells to determine the percent of cells that were BlaM positive. Images were acquired, exported as TIFF files and labeled with Adobe Illustrator.

### *C. burnetii* infections

HeLa 229 and Vero cells were plated at a density of 5×10^4^ into 24 well trays in DMEM with 2%FBS. Axenically grown *C. burnetii* strains were quantified by qPCR using *dotA* specific primers [3] to provide an accurate MOI of 50. Following a 4 h infection period, cells were washed once and incubated with fresh DMEM with 2% FBS. Wells were collected for analysis 24 (Day 1), 72 (Day 3), 120 (Day 5) and 168 (Day 7) h after this initial time point. Cells were either fixed with 4% paraformaldehyde for subsequent immunofluorescent analysis or lysed by hypotonic lysis and collected for *C. burnetii* quantification. Bacteria were pelleted from lysed cells, gDNA extracted using the illustra bacteria genomicPrep Mini Prep Kit (GE Healthcare) and genomic equivalents quantified by qPCR. To quantify the proportion of cells intracellular after the 4 h infection period fixed cells were stained with mouse anti-*C. burnetii* (1∶5000) and Alexa Fluor 596 anti-mouse before secondary fixation and permeablization. Samples were then stained with rabbit anti-*C. burnetii* (1∶10000) and Alexa Fluor 488 anti-rabbit. The proportion of cell associated bacteria that were intracellular, those stained green only, were quantified for at least 400 HeLa cells per coverslip. Two coverslips per sample were quantified from three independent experiments.

For the intracellular viability studies the infections with *C. burnetii* were performed as described above. At the indicated time points after infection the HeLa cells were extensively washed with PBS to remove any remaining extracellular bacteria before hypotonic lysis. The lysed cell material was added to 1 ml of ACCM and then diluted 1∶100 in ACCM. Genome equivalents in the starting samples were determined by qPCR. The diluted lysates in ACCM were incubated at 37°C, 5% CO_2_, 2.5% O_2_ for seven days and qPCR was used to determine the amount of replication that had occurred.

## Supporting Information

Figure S1 **Determining the ratio at which translocation of Cya-RalF by** *L. pneumophila* **can be detected in the presence of competing** *L. pneumophila* **that do not produce a Cya fusion protein having a type IV secretion signal.** *L. pneumophila* transformed with pEC33 encoding Cya alone (pCya) or pEC33 encoding Cya-RalF (pRalF) were mixed at ratios shown. Pools consisting of the bacteria at the indicated ratios (x-axis) were assayed for translocation of the Cya reporter following infection of CHO-FcγRII cells by measuring production of cAMP (y-axis). Data indicate that Dot/Icm-dependent translocation of RalF remained detectible in a mixed pool that contained 1 bacterial cell producing Cya-RalF to 49 bacterial cells containing Cya alone. Experiments were performed in triplicate and error bars represent standard deviation.
(PDF)

Figure S2 **Strategy for screening a** *C. burnetii* **library to identify clones having a Dot/Icm-dependent translocation domain fused to Cya.** (A) *C. burnetii* gene fragments were ligated at random downstream of the gene encoding Cya and the resulting plasmid library was electroporated into *L. pneumophila*. Individual *L. pneumophila* transformants were distributed into 24-well dishes to establish pools containing an estimated 50 different plasmid clones. Each pool was saved as a frozen stock, and also used to infect CHO FcγRII cells to determine whether the pool contained a potential fusion between Cya and a *C. burnetii* Dot/Icm-dependent translocation domain. The graph shows data from a screen conducted on pools of 50 clones. Four different pools (1–4) were identified that were predicted to contain clones having a Cya fusion to a Dot/Icm translocation domain based on cAMP values being 2.5-fold above background. For every positive pool, 96 single colonies were arrayed into a well in a 96-well plate. To identify single clones from a pool that had activity in the translocation assay, the plate containing clones from that pool was first screened by combining the 12 clones from each row of the plate, and testing the combined pool of 12 clones from each row for translocation activity. For any row that gave a positive signal in the translocation assay, the clones from the 12 different wells in that row were tested individually for translocation activity. (B) Shown are data obtained using the 96-well plates containing clones arrayed from pools 1–4 in panel A. An internal control for each assay was the analysis of the pool of 50 clones (grey bar). The black bars are data obtained for the pools consisting of the 12 clones from a row on the indicated plate. Highlighted are the rows from pool 1 predicted to contain positive clones (A, B), rows from pool 2 predicted to contain positive clones (C, D), and a row from pool 4 predicted to have a positive clone (E). (C) Shown are data obtained for the individual clones obtained from each positive row identified in panel B. As the data indicate, a positive clone that gave a very high translocation signal was identified from each row. Plasmid DNA was isolated from the clone in each positive well, and the fusion junction was sequenced to determine the *C. burnetii* gene attached to the Cya reporter. Multiple individual wells giving a positive signal were usually identified from the 96-well plate arrayed for a positive pool, however, the sequencing data for the clones isolated from those wells usually revealed identical fusion proteins, meaning that positive wells from a single pool typically identified siblings having the same plasmid. There were no examples where two different effectors were identified from a single pool.
(PDF)

Figure S3 **Expression of Cya fusion proteins in** *L. pneumophila* **.** Immunoblot analysis of *L. pneumophila* cell lysates indicates equal levels of protein production for the fusions Cya-CBU1780, Cya-CBU1957 and Cya-CBU2064 compared to the control proteins Cya alone and Cya-RalF. Translocation of these three Cya fusion proteins was not detected.
(PDF)

Figure S4 *Coxiella* **genes are transcribed during host cell infection.** RT-PCR analysis on bacterial RNA isolated from persistently infected CHO-FcγRII cells using primers specific for the genes indicated. (A) Analysis of *C. burnetii* genes identified initially using the Cya reporter screen. (B) Analysis of *C. burnetii* genes encoding effectors identified based on homology or proximity to other effectors. In both panels, *icmS* and *icmW* serve as positive controls. Reactions were performed in the presence (+) or absence (−) of reverse transcriptase to make certain that RNA served as the template. Locations of DNA standards are shown on the left in base pairs. Primer pairs were designed to amplify a fragment of roughly 300 bp for each gene. Shown are the results from one representative experiment out of two experiments in which similar results were obtained.
(PDF)

Figure S5 *C. burnetii* **effectors that display cytosolic localization in mammalian cells.** The indicated *C. burnetii* effector proteins tagged at the amino terminus with a 3×FLAG epitope were produced in HeLa 229 cells. Fluorescence micrographs obtained by anti-FLAG staining show diffuse cytosolic staining of each effector protein, and no difference was observed when compared to homologues encoded by the Dugway or G strains of *C. burnetii*.
(PDF)

Figure S6 **The** *icmL* **::Tn mutant has no obvious defect in replicating in defined medium or invading and surviving in cultured host cells.** (A) *Ex vivo* replication of *C. burnetii* NM (black squares) and the isogenic *icmL*::Tn mutant (white squares) was measured in ACCM. Samples were taken at the times indicated (x-axis) and genome equivalents were determined by qPCR (y-axis). The graph represents the mean ± SD from three independent experiments. (B) *C. burnetii* NM and the isogenic *icmL*::Tn mutant were added to HeLa cells at a multiplicity of infection of 50 and incubated for 4 h at 37°C. Cells were washed and fixed with 4% paraformaldehyde. Before cells were permeabilized, extracellular *C. burnetii* were stained with a mouse anti-*C. burnetii* antibody (1∶5000) and an Alexa Fluor 596-labeled anti-mouse secondary antibody. After a second round of fixation, cells were permeabilized and stained with a rabbit anti-*C. burnetii* antibody (1∶10000) and an Alexa Fluor 488-labeled anti-rabbit secondary antibody. Cells were visualized by fluorescence microscopy and differential staining of intracellular and extracellular bacteria was used to determine the ratio of intracellular and extracellular *C. burnetii* by counting bacteria associated with at least 400 HeLa cells on a coverslip. Two coverslips were examined for each strain in three independent experiments. Data are presented as the percent of cell-associated *C. burnetii* that were intracellular (x-axis) for the NM strain (grey bar) and the *icmL*::Tn mutant (white bar). Values are the mean ± SD for the three independent experiments. No significant difference was observed for the internalization of the NM strain compared to the *icmL*::Tn mutant. (C) HeLa cells infected at a MOI of 50 were extensively washed and lysed at the indicated times. The samples were diluted 1∶100 in ACCM and incubated for 7 days. The 1∶100 dilutions showed signs of bacterial replication for all samples, suggesting the presence of viable bacteria in the samples obtained from infected host cells. *C. burnetii* GE were calculated in the 1∶00 dilutions after 7 days of incubation and plotted as fold increase compared to the GE number obtained for that dilution at the time of cell lysis. *C. burnetii* NM (black bars), *C. burnetii* NM treated with 10 µg/ml chloramphenicol (grey bars) and *icmL*::Tn (white bars) showed similar growth after extraction from HeLa cells indicating the comparable viability of intracellular bacteria. This experiment yielded similar results when conducted for duplicate wells in three independent infections.
(PDF)

Figure S7 **Expression of BlaM-effector fusion proteins in** *C. burnetii* **.** Immunoblot analysis of *C. burnetii* NM phase II and the *icmL*::Tn mutant harboring pJB-CAT-BlaM (BlaM) and BlaM-effector fusion constructs (BlaM-77, BlaM-0635 and BlaM-1524). Probing equivalent amounts of these *C. burnetii* transformants with anti-BlaM demonstrated expression of BlaM (29 kDa), BlaM-77 (60 kDa), BlaM-0635 (84 kDa) and BlaM-1524 (54 kDa). Importantly, expression of each reporter protein was comparable in *C. burnetii* NM phase II and the *icmL*::Tn mutant.
(PDF)

Table S1 **Cya fusions screened that were positive for protein translocation.** Indicated are the proteins identified in the screen for *C. burnetii* proteins with a Dot/Icm-dependent translocation signal. Shown are the proteins identified, the number of times each protein was identified in the screen, and the position in the location of the Cya fusion junction in the predicted protein product.
(DOC)

Table S2 **Additional** *C. burnetii* **genes examined for Dot/Icm-dependent translocation.** Indicated are the *C. burnetii* genes with homology or proximity to the genes encoding proteins identified in the screen for Dot/Icm-dependent translocation signals. The genes were fused to Cya to test for Dot/Icm-dependent translocation, and the translocation results are given.
(DOC)

Table S3 **Primers used in this study.** Listed are the sequences of primers used in this study to create plasmids encoding the *C. burnetii* effectors, to test for their expression, and to map the location of transposon insertions. Restriction sites in primers that were used to ligate the resulting PCR products into plasmids are underlined.
(DOC)
