# Supplementary figures and images for: The Coxiella burnetii Dot/Icm System Delivers a Unique Repertoire of Type IV Effectors into Host Cells and Is Required for Intracellular Replication
Source: PLoS Pathog. 2011 May 26;7(5):e1002056. doi: 10.1371/journal.ppat.1002056 (PMC3102713; doi:10.1371/journal.ppat.1002056)

Supplementary Figure 1

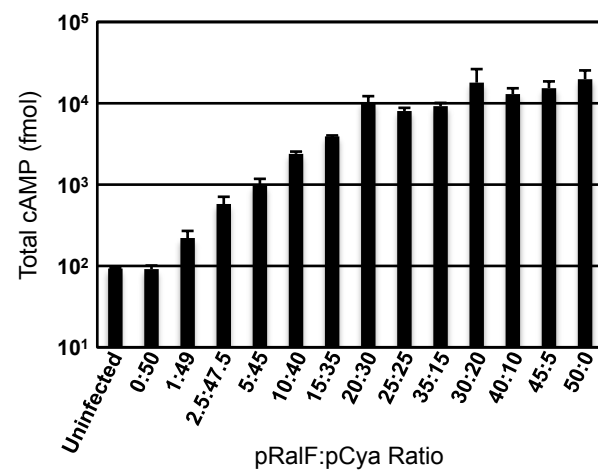

Supplement: Figure S1 — Determining the ratio at which translocation of Cya-RalF by L. pneumophila can be detected in the presence of competing L. pneumophila that do not produce a Cya fusion protein having a type IV secretion signal. L. pneumophila transformed with pEC33 encoding Cya alone (pCya) or pEC33 encoding Cya-RalF (pRalF) were mixed at ratios shown. Pools consisting of the bacteria at the indicated ratios (x-axis) were assayed for translocation of the Cya reporter following infection of CHO-FcγRII cells by measuring production of cAMP (y-axis). Data indicate that Dot/Icm-dependent translocation of RalF remained detectible in a mixed pool that contained 1 bacterial cell producing Cya-RalF to 49 bacterial cells containing Cya alone. Experiments were performed in triplicate and error bars represent standard deviation. (PDF) [file ppat.1002056.s001.pdf]

## Supplementary Figure 2

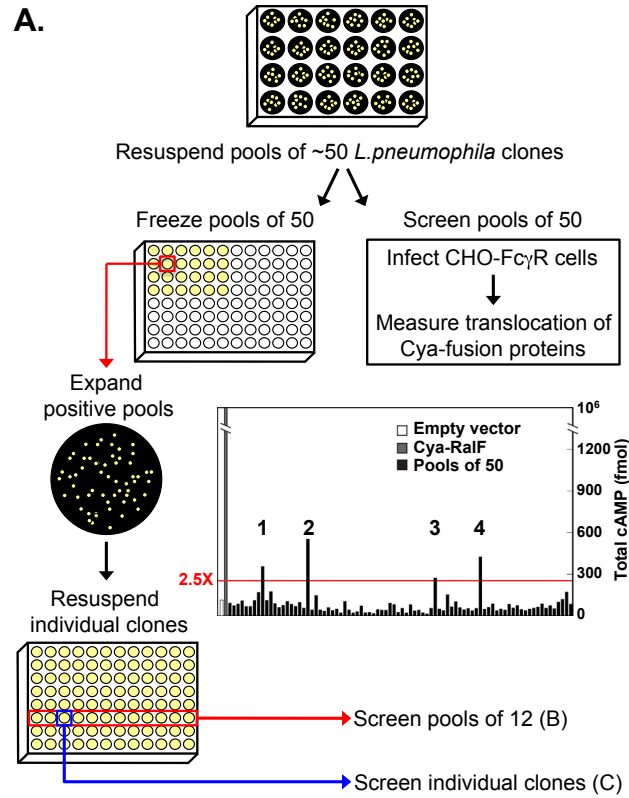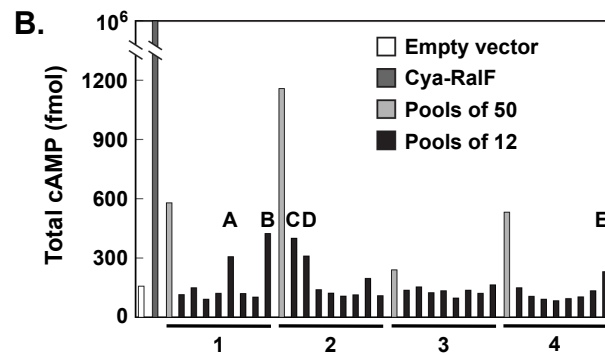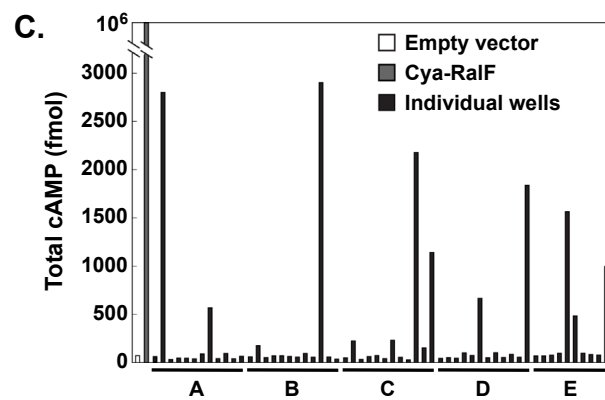

Supplement: Figure S2 — Strategy for screening a C. burnetii library to identify clones having a Dot/Icm-dependent translocation domain fused to Cya. (A) C. burnetii gene fragments were ligated at random downstream of the gene encoding Cya and the resulting plasmid library was electroporated into L. pneumophila. Individual L. pneumophila transformants were distributed into 24-well dishes to establish pools containing an estimated 50 different plasmid clones. Each pool was saved as a frozen stock, and also used to infect CHO FcγRII cells to determine whether the pool contained a potential fusion between Cya and a C. burnetii Dot/Icm-dependent translocation domain. The graph shows data from a screen conducted on pools of 50 clones. Four different pools (1–4) were identified that were predicted to contain clones having a Cya fusion to a Dot/Icm translocation domain based on cAMP values being 2.5-fold above background. For every positive pool, 96 single colonies were arrayed into a well in a 96-well plate. To identify single clones from a pool that had activity in the translocation assay, the plate containing clones from that pool was first screened by combining the 12 clones from each row of the plate, and testing the combined pool of 12 clones from each row for translocation activity. For any row that gave a positive signal in the translocation assay, the clones from the 12 different wells in that row were tested individually for translocation activity. (B) Shown are data obtained using the 96-well plates containing clones arrayed from pools 1–4 in panel A. An internal control for each assay was the analysis of the pool of 50 clones (grey bar). The black bars are data obtained for the pools consisting of the 12 clones from a row on the indicated plate. Highlighted are the rows from pool 1 predicted to contain positive clones (A, B), rows from pool 2 predicted to contain positive clones (C, D), and a row from pool 4 predicted to have a positive clone (E). (C) Shown are data obtained for the i [file ppat.1002056.s002.pdf]

Supplementary Figure 3:

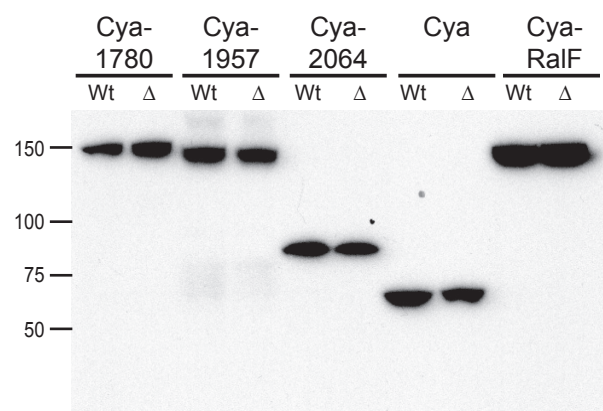

Supplement: Figure S3 — Expression of Cya fusion proteins in L. pneumophila . Immunoblot analysis of L. pneumophila cell lysates indicates equal levels of protein production for the fusions Cya-CBU1780, Cya-CBU1957 and Cya-CBU2064 compared to the control proteins Cya alone and Cya-RalF. Translocation of these three Cya fusion proteins was not detected. (PDF) [file ppat.1002056.s003.pdf]

Supplementary Figure 4

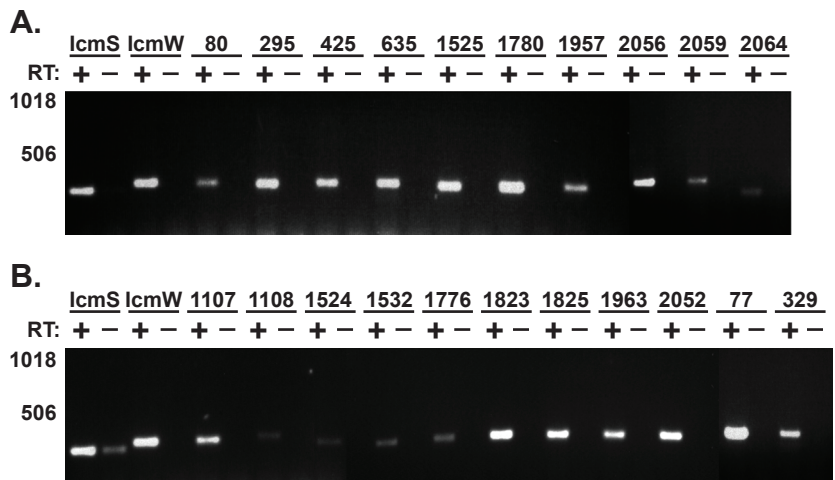

Supplement: Figure S4 — Coxiella genes are transcribed during host cell infection. RT-PCR analysis on bacterial RNA isolated from persistently infected CHO-FcγRII cells using primers specific for the genes indicated. (A) Analysis of C. burnetii genes identified initially using the Cya reporter screen. (B) Analysis of C. burnetii genes encoding effectors identified based on homology or proximity to other effectors. In both panels, icmS and icmW serve as positive controls. Reactions were performed in the presence (+) or absence (−) of reverse transcriptase to make certain that RNA served as the template. Locations of DNA standards are shown on the left in base pairs. Primer pairs were designed to amplify a fragment of roughly 300 bp for each gene. Shown are the results from one representative experiment out of two experiments in which similar results were obtained. (PDF) [file ppat.1002056.s004.pdf]

Supplementary Figure 5

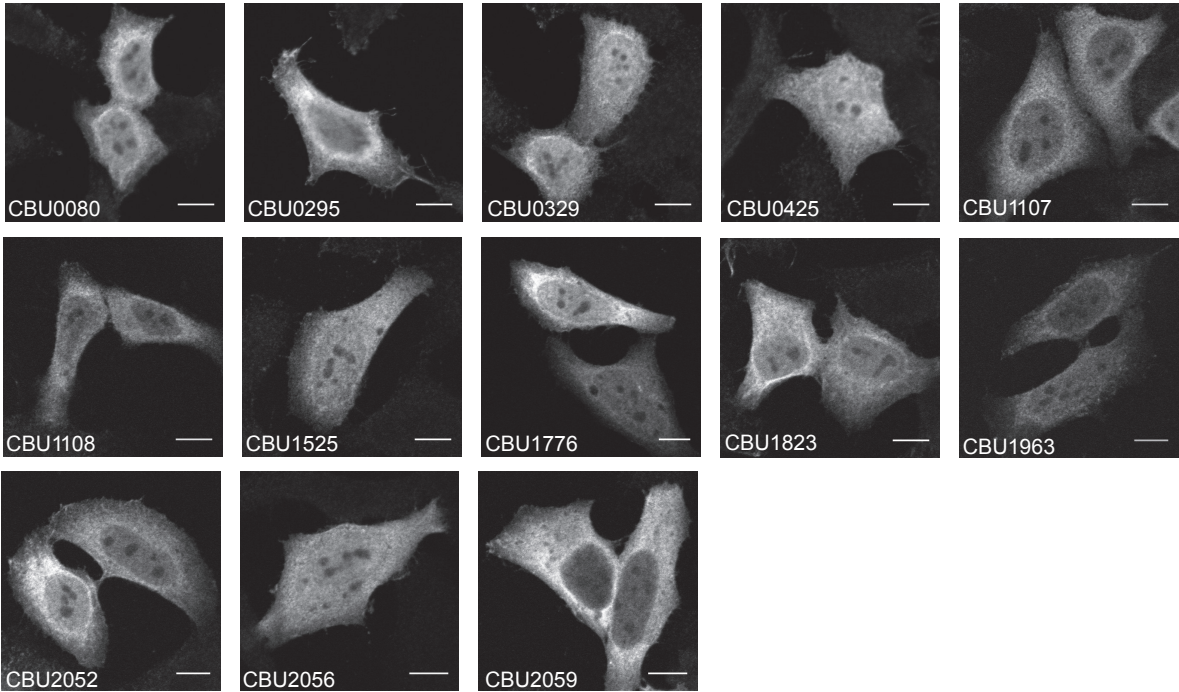

Supplement: Figure S5 — C. burnetii effectors that display cytosolic localization in mammalian cells. The indicated C. burnetii effector proteins tagged at the amino terminus with a 3×FLAG epitope were produced in HeLa 229 cells. Fluorescence micrographs obtained by anti-FLAG staining show diffuse cytosolic staining of each effector protein, and no difference was observed when compared to homologues encoded by the Dugway or G strains of C. burnetii. (PDF) [file ppat.1002056.s005.pdf]

Supplementary Figure 6:

**A**

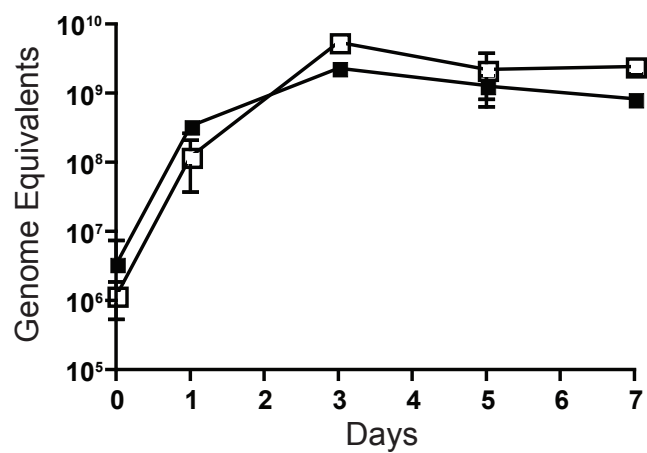

**B**

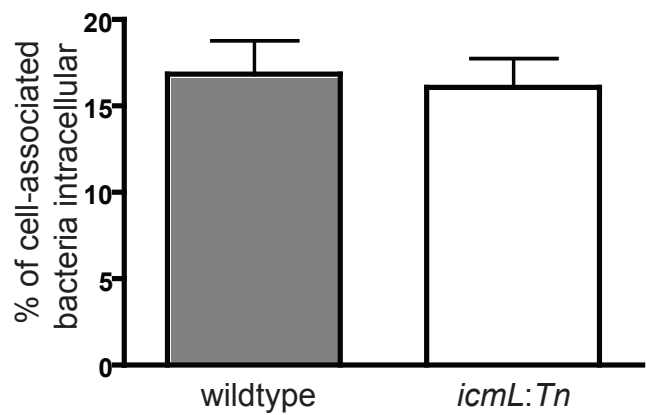

**C**

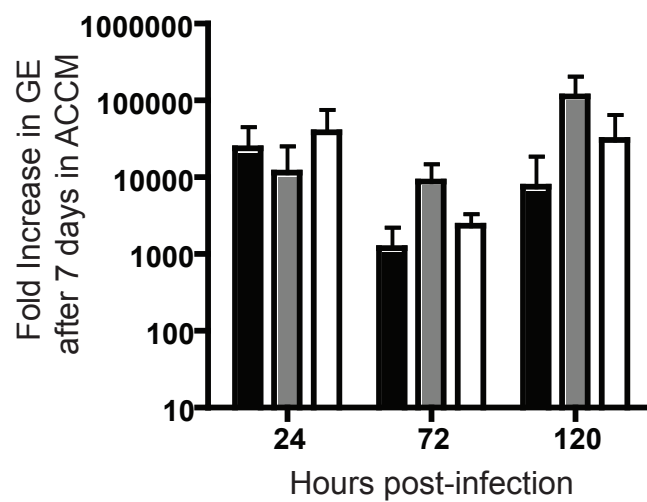

Supplement: Figure S6 — The icmL ::Tn mutant has no obvious defect in replicating in defined medium or invading and surviving in cultured host cells. (A) Ex vivo replication of C. burnetii NM (black squares) and the isogenic icmL::Tn mutant (white squares) was measured in ACCM. Samples were taken at the times indicated (x-axis) and genome equivalents were determined by qPCR (y-axis). The graph represents the mean ± SD from three independent experiments. (B) C. burnetii NM and the isogenic icmL::Tn mutant were added to HeLa cells at a multiplicity of infection of 50 and incubated for 4 h at 37°C. Cells were washed and fixed with 4% paraformaldehyde. Before cells were permeabilized, extracellular C. burnetii were stained with a mouse anti-C. burnetii antibody (1∶5000) and an Alexa Fluor 596-labeled anti-mouse secondary antibody. After a second round of fixation, cells were permeabilized and stained with a rabbit anti-C. burnetii antibody (1∶10000) and an Alexa Fluor 488-labeled anti-rabbit secondary antibody. Cells were visualized by fluorescence microscopy and differential staining of intracellular and extracellular bacteria was used to determine the ratio of intracellular and extracellular C. burnetii by counting bacteria associated with at least 400 HeLa cells on a coverslip. Two coverslips were examined for each strain in three independent experiments. Data are presented as the percent of cell-associated C. burnetii that were intracellular (x-axis) for the NM strain (grey bar) and the icmL::Tn mutant (white bar). Values are the mean ± SD for the three independent experiments. No significant difference was observed for the internalization of the NM strain compared to the icmL::Tn mutant. (C) HeLa cells infected at a MOI of 50 were extensively washed and lysed at the indicated times. The samples were diluted 1∶100 in ACCM and incubated for 7 days. The 1∶100 dilutions showed signs of bacterial replication for all samples, suggesting the presence of viable bacteria in the samples obtained fr [file ppat.1002056.s006.pdf]

Supplementary Figure 7

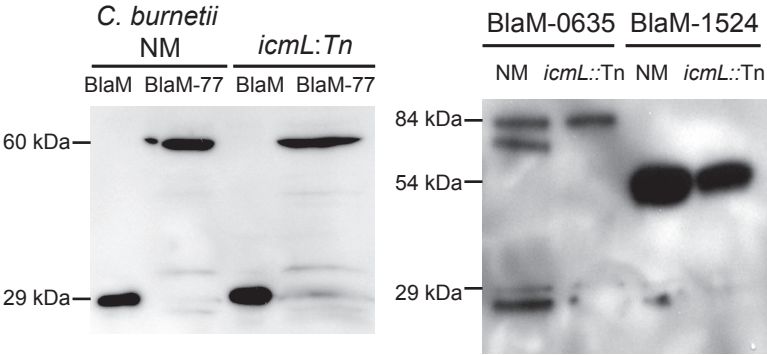

Supplement: Figure S7 — Expression of BlaM-effector fusion proteins in C. burnetii . Immunoblot analysis of C. burnetii NM phase II and the icmL::Tn mutant harboring pJB-CAT-BlaM (BlaM) and BlaM-effector fusion constructs (BlaM-77, BlaM-0635 and BlaM-1524). Probing equivalent amounts of these C. burnetii transformants with anti-BlaM demonstrated expression of BlaM (29 kDa), BlaM-77 (60 kDa), BlaM-0635 (84 kDa) and BlaM-1524 (54 kDa). Importantly, expression of each reporter protein was comparable in C. burnetii NM phase II and the icmL::Tn mutant. (PDF) [file ppat.1002056.s007.pdf]
