# Supplementary material for: The Coxiella burnetii Dot/Icm System Delivers a Unique Repertoire of Type IV Effectors into Host Cells and Is Required for Intracellular Replication
Source: PLoS Pathog. 2011 May 26;7(5):e1002056. doi: 10.1371/journal.ppat.1002056 (PMC3102713; doi:10.1371/journal.ppat.1002056)
Supplement: Table S1 — Cya fusions screened that were positive for protein translocation. Indicated are the proteins identified in the screen for C. burnetii proteins with a Dot/Icm-dependent translocation signal. Shown are the proteins identified, the number of times each protein was identified in the screen, and the position in the location of the Cya fusion junction in the predicted protein product. (DOC) [file ppat.1002056.s008.doc]

**Table S1.** Cya fusions that screened positive for protein translocation.

| Translocated protein | Number of times hit | Junction site |
| --- | --- | --- |
| CBU0080 | 1 | 15 amino acids upstream of ATG |
| CBU0295 | 2 | Amino acid 295 |
| CBU0425 | 2 | Amino acid 407 |
| CBU0635 | 2 | Amino acid 470 |
| CBU1525 | 1 | Amino acid 2 |
| CBU1780 | 1 | Amino acid 206 |
| CBU1957 | 1 | Amino acid 69 |
| CBU2056 | 1 | Amino acid 76 |
| CBU2059 | 2 | Amino acid 257 |
| CBU2064 | 2 | Amino acid 42 |
