# Supplementary material for: The Coxiella burnetii Dot/Icm System Delivers a Unique Repertoire of Type IV Effectors into Host Cells and Is Required for Intracellular Replication
Source: PLoS Pathog. 2011 May 26;7(5):e1002056. doi: 10.1371/journal.ppat.1002056 (PMC3102713; doi:10.1371/journal.ppat.1002056)
Supplement: Table S2 — Additional C. burnetii genes examined for Dot/Icm-dependent translocation. Indicated are the C. burnetii genes with homology or proximity to the genes encoding proteins identified in the screen for Dot/Icm-dependent translocation signals. The genes were fused to Cya to test for Dot/Icm-dependent translocation, and the translocation results are given. (DOC) [file ppat.1002056.s009.doc]

**Table S2. Additional *C. burnetii* genes examined for Dot/Icm-dependent translocation.**

| Gene | Reason for testing | Translocation by *L. pneumophila* |
| --- | --- | --- |
| CBU0077 | Upstream of CBU0080 | Yes |
| CBU0322 | Upstream of CBU0328 and CBU0329 | No |
| CBU0328 | Homology to CBU1525 | No |
| CBU0329 | Downstream of CBU0328 | Yes |
| CBU0339 | Downstream of CBU0328 and CBU0329 | No |
| CBU0340 | Downstream of CBU0328 and CBU0329 | No |
| CBU0632 | Upstream of CBU0653 | No |
| CBU1105 | Upstream of CBU1108 | No |
| CBU1106 | Upstream of CBU1108 | No |
| CBU1107 | Upstream of CBU1108 | Yes |
| CBU1108 | Homology to CBU1525 | Yes |
| CBU1109 | Downstream of CBU1108 | No |
| CBU1454 | Homology to CBU1108 | No |
| CBU1524 | Upstream of CBU1525 | Yes |
| CBU1526 | Downstream of CBU1525 | No |
| CBU1527 | Downstream of CBU1525 | No |
| CBU1528 | Downstream of CBU1525 | No |
| CBU1530 | Downstream of CBU1525 | No |
| CBU1531 | Downstream of CBU1525 | No |
| CBU1532 | Downstream of CBU1525 | Yes |
| CBU1533 | Downstream of CBU1525 | No |
| CBU1535 | Downstream of CBU1525 | No |
| CBU1762 | Upstream of CBU1780 | No |
| CBU1768 | Upstream of CBU1780 | No |
| CBU1774 | Upstream of CBU1780 | No |
| CBU1775 | Upstream of CBU1780 | No |
| CBU1776 | Upstream of CBU1780 | Yes |
| CBU1823 | Homology to CBU1963 | Yes |
| CBU1825 | Homology to CBU1823 | Yes |
| CBU1953 | Upstream of CBU1957 | No |
| CBU1963 | Downstream of CBU1957 | Yes |
| CBU2051 | Proximity to CBU2056 and CBU2059 | No |
| CBU2052 | Proximity to CBU2056 and CBU2059 | Yes |
| CBU2057 | Proximity to CBU2056 and CBU2059 | No |
| CBU2062 | Upstream of CBU2064 | No |
| CBU2063 | Upstream of CBU2064 | No |
| CBU2065 | Downstream of CBU2064 | No |
