# Supplementary material for: The Coxiella burnetii Dot/Icm System Delivers a Unique Repertoire of Type IV Effectors into Host Cells and Is Required for Intracellular Replication
Source: PLoS Pathog. 2011 May 26;7(5):e1002056. doi: 10.1371/journal.ppat.1002056 (PMC3102713; doi:10.1371/journal.ppat.1002056)
Supplement: Table S3 — Primers used in this study. Listed are the sequences of primers used in this study to create plasmids encoding the C. burnetii effectors, to test for their expression, and to map the location of transposon insertions. Restriction sites in primers that were used to ligate the resulting PCR products into plasmids are underlined. (DOC) [file ppat.1002056.s010.doc]

Table S3. Primers used in this study.

| Primer | Sequence 5’ to 3’ | Purpose |
| --- | --- | --- |
| CBU0077 F1 | AAGGATCCTTATGAGACAACTCGTTTCAATTAA | pEC33 |
| CBU0077 R1 | AACTGCAGTTACATAATAGAACACCCACGAC | pEC33 |
| CBU0080 F1 | AAGGATCCTTTTGGGTCATCGTGAGAAAGAA | pEC33 |
| CbuK1976 F1 | AAGGATCCATGAGGCACGAAAATCCCCA | pcDNA4/T 3xFLAG |
| CBU0080 R1 | AACTGCAGTTATTGAAATGCTCTCGTCTGG | pEC33 |
| CBU0295 F1 | AAGGATCCTTTTGAGGTACAAGCGACACATG | pEC33 |
| CBU0295 R1 | AACTGCAGTTAAAAAGTAAAGGATTGTTTAGAG | pEC33 |
| CBU0322 F1 | AAGGATCCTTATGTCTGACTCTCCATTCATAC | pEC33 |
| CBU0322 R1 | AACTGCAGTCATGATAATACCTGACGAAAAT | pEC33 |
| CBU0328 F1 | AAGGATCCTTGTGATGATAAATTATGATAGATTG | pEC33 |
| CBU0328 R1 | AACTGCAGTCAATATTTTATAGCTGGCACGA | pEC33 |
| CBU0329 F1 | AAGGATCCTTATGGAGCTTTGTGCCAGTGA | pEC33 |
| CBUD1750 F1 | AAGGATCCATGGTTGCAAAAAAAGAGTGTG | pcDNA4/T 3xFLAG |
| CBU0329 R1 | AACTGCAGTTATCTGCGTTGCAATCGGG | pEC33 |
| CBU0339 F1 | AAGGATCCTTATGCGAAAATCCCACCCCAC | pEC33 |
| CBU0339 R1 | AACTGCAGTTAAGCTTGAAAAAATTGATAATCT | pEC33 |
| CBU0340 F1 | AAGGATCCTTATGGCAGTTGTCTATCAATCAA | pEC33 |
| CBU0340 R1 | AACTGCAGTTAATCGTGAAATACCTCTTCTA | pEC33 |
| CBU0425 F1 | AAGGATCCTTATGGAAGGCGTAATCTATTCAC | pEC33 |
| CbuG1587 F1 | AAGGATCCATGAATCAACAACAACGAATAC | pcDNA4/T 3xFLAG |
| CBU0425 R1 | AACTGCAGTTATGTGGATGCGCTGGGC | pEC33 |
| CBU0632 F1 | AAGGATCCTTATGACTAGCGAAAATTACCATG | pEC33 |
| CBU0632 R1 | AACTGCAGTTATTCACCCGAGTGGTGATC | pEC33 |
| CBU0635 F1 | AAGGATCCTTATGCGAGAGGAAAAAGAGGAG | pEC33 |
| CBU0635 R1 | AACTGCAGCTAAACTAATGTCATTAAACGGT | pEC33 |
| CBU1105 F1 | AAGGATCCTTATGCGGAAAAGAGATGAAAATAT | pEC33 |
| CBU1105 R1 | AACTGCAGTTAAAAAGCACCGCCGATTTTC | pEC33 |
| CBU1106 F1 | AAGGATCCTTATGACAATATCTAATAATCCCAAA | pEC33 |
| CBU1106 R1 | AACTGCAGTTAAGCGTTTGCTAGCATTAAAT | pEC33 |
| CBU1107 F1 | AAGGATCCTTATGAAGGTGTCCCAATGTGG | pEC33 |
| CBUD1210 F1 | AAGGATCCTTGAATAAATTAGGGTCTATTTAC | pcDNA4/T 3xFLAG |
| CBU1107 R1 | AACTGCAGTTATCCAGGTTGTCTATTAAATTG | pEC33 |
| CBU1108 F1 | AAGGATCCTTTTGTTATATTCCATTTTTAAGGCG | pEC33 |
| CBU1108 R1 | AACTGCAGCTATTTTGTGTAATTGATGAAAAAG | pEC33 |
| CBU1109 F1 | AAGGATCCTTGTGAAAAGCGAAGAGCTATTC | pEC33 |
| CBU1109 R1 | AACTGCAGTTAATTCATCATAATAATATTCATTC | pEC33 |
| CBU1454 F1 | AAGGATCCTTATGCTTAGCCTTTCCAACCAG | pEC33 |
| CBU1454 R1 | AACTGCAGTCACCGCCATCTCTTTTTGG | pEC33 |
| CBU1524 F1 | AAGGATCCTTATGAACACAAGTCCTACATCAA | pEC33 |
| CBUD0462 F1 | AAGGATCCATGAGAAAAAAACCTAACGTCG | pcDNA4/T 3xFLAG |
| CBU1524 R1 | AACTGCAGCTATGTCCTTTTGGGAGCGT | pEC33 |
| CBU1525 F1 | AAGGATCCTTTTGATCAGCGATTTTACTTCGG | pEC33 |
| CBUD0461 F1 | AAGGATCCATGTCCCGTCGCGAGCCT | pcDNA4/T 3xFLAG |
| CBU1525 R1 | AACTGCAGTTAAATCGTAAGGCGCTTTGTT | pEC33 |
| CBU1526 F1 | AAGGATCCTTATGTCCCGTCGCGAGCCT | pEC33 |
| CBU1526 R1 | AACTGCAGTTAAAAAATTAAAAATACTCGGCAA | pEC33 |
| CBU1527 F1 | AAGGATCCTTATGCTTCTCTTTTTATCACTTATT | pEC33 |
| CBU1527 R1 | AACTGCAGTTAGGCATTATAAGGGCGATTT | pEC33 |
| CBU1528 F1 | AAGGATCCTTTTGCTAAAGGATGTCACCAAC | pEC33 |
| CBU1528 R1 | AACTGCAGTTAATTTAAGATTTTTTCGATATTGA | pEC33 |
| CBU1530 F1 | CAAGTCGACTATGCCTTTATCTAAAGAAGAATTC | pEC33 |
| CBU1530 R1 | AAGCATGCTTAAATTAGTCTAGCTCGGAGA | pEC33 |
| CBU1531 F1 | AAGGATCCTTTTGCGAGACGAGAACGATCC | pEC33 |
| CBU1531 R1 | AACTGCAGCTAATTTTTTAGCACGTCTTTTTT | pEC33 |
| CBU1532 F1 | AAGGATCCTTTTGGCAGGCATAGCTGCAAC | pEC33 |
| CBUD0454 F1 | AAGGATCCTTGCGAGACGAGAACGATCC | pcDNA4/T 3xFLAG |
| CBU1532 R1 | AACTGCAGTTACTTATTAAATTCGGGTATGTA | pEC33 |
| CBU1533 F1 | CAAGTCGACTATGATGCCCGCTGAGCTCG | pEC33 |
| CBU1533 R1 | AAGCATGCTCAAGGCGCCAGGTTAGAGG | pEC33 |
| CBU1535 F1 | AAGGATCCTTATGCGATTCAATATGGCTATTG | pEC33 |
| CBU1535 R1 | AACTGCAGTTAGGAATTGCTAACAAATAATGC | pEC33 |
| CBU1762 F1 | AAGGATCCTTATGAAAACTCAACTATCCGCAG | pEC33 |
| CBU1762 R1 | AACTGCAGTCAAGCGCACCCTCTTTTTAC | pEC33 |
| CBU1768 F1 | AAGGATCCTTATGTATAAATTGTTAAGTTATCCTT | pEC33 |
| CBU1768 R1 | AACTGCAGTTATTTATGAATGAACTGACAGTT | pEC33 |
| CBU1774 F1 | AAGGATCCTTGTGAATGATCAACGATCTCTG | pEC33 |
| CBU1774 R1 | AACTGCAGTCACAAAGCTTGCGGCGCG | pEC33 |
| CBU1775 F1 | AAGGATCCTTATGCCATTAAAAGACTTACGCG | pEC33 |
| CBU1775 R1 | AACTGCAGTTACGCATTTTCAAGGATGGG | pEC33 |
| CBU1776 F1 | AAGGATCCTTTTGTATATTAACATCAATCAATCC | pEC33 |
| CBUD0231 F1 | AAGGATCCATGCCATTAAAAGACTTACGC | pcDNA4/T 3xFLAG |
| CBU1776 R1 | AACTGCAGTTACTTTATAAAAGTCCGTGTATA | pEC33 |
| CBU1823 F1 | AAGGATCCTTATGCCTAAACTCAGTAACCGT | pEC33 |
| CBU1823 R1 | AACTGCAGTTATGGCCTCTTATTTGTTGGC | pEC33 |
| CBU1825 F1 | AAGGATCCTTATGTTGGTTAGTAATACCAGCA | pEC33 |
| CBUD0054 F1 | AAGGATCCGTGTATGCAAACCATTTGATAC | pcDNA4/T 3xFLAG |
| CBU1825 R1 | AACTGCAGCTATTTTTTTGTCATTTCCAGATT | pEC33 |
| CBU1953 F1 | AAGGATCCTTATGTTTTCTTATTTAAGTACCTGT | pEC33 |
| CBU1953 R1 | AACTGCAGTCAAGGGAGAGTAGATTGTTC | pEC33 |
| CBU1963 F1 | AAGGATCCTTATGGAGTTTCTAATAAAGTTTTCT | pEC33 |
| CBUD2063 F1 | AAGGATCCATGCGAGTTGAGCTGTGGC | pcDNA4/T 3xFLAG |
| CBU1963 R1 | AACTGCAGTCAAGGATGATGGTGGCGAG | pEC33 |
| CBU2051 F1 | AAGGATCCTTATGCCCAATTGTACGAACGAA | pEC33 |
| CBU2051 R1 | AACTGCAGTCATGTGTTCGTTGTACGACG | pEC33 |
| CBU2052 F1 | AAGGATCCTTATGCCTAAAAACACAAATCCAG | pEC33 |
| CBU2052 R1 | AACTGCAGTTATTTCAAAAAAGCATTTACAAGA | pEC33 |
| CBU2056 F1 | AAGGATCCTTGTGAGTTTAATTTTCATAAAGATTC | pEC33 |
| CBUD2151 F1 | AAGGATCCATGTCTCGTGGTGAGTTTAATT | pcDNA4/T 3xFLAG |
| CBU2056 R1 | AACTGCAGCTAAGGTGCGGGTGCGCG | pEC33 |
| CBU2057 F1 | AAGGATCCTTATGGCAGCCGTCCATCAATC | pEC33 |
| CBU2057 R1 | AACTGCAGTTAATCTTGAAATATCTCTTCTAAA | pEC33 |
| CBU2059 F1 | AAGGATCCTTGTGTCGGAACCCCCGAATAA | pEC33 |
| CBUD2154 F1 | AAGGATCCATGACAAGACAAACAACTGAAA | pcDNA4/T 3xFLAG |
| CBU2059 R1 | AACTGCAGTCATTTGATTGTTAAGGAAGAAG | pEC33 |
| CBU2062 F1 | AAGGATCCTTATGCCCGTTTCTTTTTGTTCTA | pEC33 |
| CBU2062 R1 | AACTGCAGTCAAATATTCCATAGCAAAATTCC | pEC33 |
| CBU2063 F1 | AAGGATCCTTATGGAATTTTGCTATGGAATATTT | pEC33 |
| CBU2063 R1 | AACTGCAGTCATTTGTTGAAGAATAAACAGC | pEC33 |
| CBU2065 F1 | AAGGATCCTTATGTCTTTTTTAGCTATTAATGTC | pEC33 |
| CBU2065 R1 | AACTGCAGTTAAAATTTCCACCATGGTTTTTG | pEC33 |
| IcmS RTF | CCAACTTCACCTTGAAAGGCC | RT-PCR [9] |
| IcmS RTR | CGTACATCAGTTCATCCAGCG | RT-PCR [9] |
| IcmW RTF | TAAAGCCGTCCATCAATTTTGG | RT-PCR [9] |
| IcmW RTR | GCGGTGTCTATTTCCGATTTTG | RT-PCR [9] |
| CBU0077 RTF | ATGAGACAACTCGTTTCAATTAA | RT-PCR |
| CBU0077 RTR | CTGCGCTCCTATTTCCTGC | RT-PCR |
| CBU0080 RTF | TTGGGTCATCGTGAGAAAGAA | RT-PCR |
| CBU0080 RTR | CCGCTTTGCAAACTATAACTTG | RT-PCR |
| CBU0295 RTF | TTGAGGTACAAGCGACACATG | RT-PCR |
| CBU0295 RTR | GACCGGTATTTTGTTTAACTCC | RT-PCR |
| CBU0329 RTF | ATGGAGCTTTGTGCCAGTGA | RT-PCR |
| CBU0329 RTR | TGAAACAAATAATCCATTTAATACT | RT-PCR |
| CBU0425 RTF | ATGGAAGGCGTAATCTATTCAC | RT-PCR |
| CBU0425 RTR | CCTTCATAAAATGAGGTGCGC | RT-PCR |
| CBU0635 RTF | ATGCGAGAGGAAAAAGAGGAG | RT-PCR |
| CBU0635 RTR | GAATCACCGAAAGAAACGTCT | RT-PCR |
| CBU1107 RTF | ATGAAGGTGTCCCAATGTGG | RT-PCR |
| CBU1107 RTR | CTTCTTCTTCTGAACATAAGTG | RT-PCR |
| CBU1108 RTF | TTGTTATATTCCATTTTTAAGGCG | RT-PCR |
| CBU1108 RTR | CTTATCATATTGATTAAATCAAGC | RT-PCR |
| CBU1524 RTF | ATGAACACAAGTCCTACATCAA | RT-PCR |
| CBU1524 RTR | GTGCGTTAGTGTGCTTATCTC | RT-PCR |
| CBU1525 RTF | TTGATCAGCGATTTTACTTCGG | RT-PCR |
| CBU1525 RTR | GACTCAATAAACTCTTAAGTTCT | RT-PCR |
| CBU1532 RTF | TTGGCAGGCATAGCTGCAAC | RT-PCR |
| CBU1532 RTR | CTAAAGCAACGATACGAGCG | RT-PCR |
| CBU1776 RTF | TTGTATATTAACATCAATCAATCC | RT-PCR |
| CBU1776 RTR | GTGAAGAGGGTCTCGCATC | RT-PCR |
| CBU1780 RTF | GTGGGAAATACTTCGGTTATTT | RT-PCR |
| CBU1780 RTR | CGAAAGTTAGCTCGTGTTGTACC | RT-PCR |
| CBU1823 RTF | ATGCCTAAACTCAGTAACCGT | RT-PCR |
| CBU1823 RTR | GCACGATAGAACATTGTGATC | RT-PCR |
| CBU1825 RTF | ATGTTGGTTAGTAATACCAGCA | RT-PCR |
| CBU1825 RTR | GGTTATCAGTTTTAGGCTCATC | RT-PCR |
| CBU1957 RTF | GTGATCGGTTTCATTCATTTAAG | RT-PCR |
| CBU1957 RTR | CTCGAACTTAGCGTTTCACTC | RT-PCR |
| CBU1963 RTF | ATGGAGTTTCTAATAAAGTTTTCT | RT-PCR |
| CBU1963 RTR | GCGGTAGAAAGAGTCTCTCT | RT-PCR |
| CBU2052 RTF | ATGCCTAAAAACACAAATCCAG | RT-PCR |
| CBU2052 RTR | CCTTCAAAGAAAGTACTTTAAAC | RT-PCR |
| CBU2056 RTF | GTGAGTTTAATTTTCATAAAGATTC | RT-PCR |
| CBU2056 RTR | CCGTATCCGTTTTATTTTCTTC | RT-PCR |
| CBU2059 RTF | GTGTCGGAACCCCCGAATA | RT-PCR |
| CBU2059 RTR | ACGTTCACTCTCCACTTTTTC | RT-PCR |
| CBU2064 RTF | TTGCTGTTTATTCTTCAACAAATG | RT-PCR |
| CBU2064 RTR | TTATCGGTTCAGGTTGCTACT | RT-PCR |
| CBU0077 F2 | AAGGATCCAACACAATGAGACAACTCGTTTCAATTAA | pYES2 |
| CBU0077 R2 | GCTCTAGATTACATAATAGAACACCCACGA | pYES2 |
| CBU0080 F2 | AAGGATCCAACACAATGGGTCATCGTGAGAAAGAA | pYES2 |
| CBU0080 R2 | GCTCTAGATTATTGAAATGCTCTCGTCTGG | pYES2 |
| CBU0295 F2 | AAGGATCCAACACAATGAGGTACAAGCGACACATG | pYES2 |
| CBU0295 R2 | GCTCTAGATTAAAAAGTAAAGGATTGTTTAGAG | pYES2 |
| CBU0329 F2 | AAGGATCCAACACAATGGAGCTTTGTGCCAGTG | pYES2 |
| CBU0329 R2 | GCTCTAGATTATCTGCGTTGCAATCGGGA | pYES2 |
| CBU0425 F2 | AAGGATCCAACACAATGGAAGGCGTAATCTATTCAC | pYES2 |
| CBU0425 R2 | GCTCTAGATTATGTGGATGCGCTGGGC | pYES2 |
| CBU0635 F2 | AAGGATCCAACACAATGCGAGAGGAAAAAGAGGAG | pYES2 |
| CBU0635 R2 | GCTCTAGACTAAACTAATGTCATTAAACGGTT | pYES2 |
| CBU1107 F2 | AAGGATCCAACACAATGAAGGTGTCCCAATGTGG | pYES2 |
| CBU1107 R2 | GCTCTAGATTATCCAGGTTGTCTATTAAATTG | pYES2 |
| CBU1108 F2 | AAGGATCCAACACAATGTTATATTCCATTTTTAAGGCG | pYES2 |
| CBU1108 R2 | GCTCTAGACTATTTTGTGTAATTGATGAAAAAG | pYES2 |
| CBU1524 F2 | AAGGATCCAACACAATGAACACAAGTCCTACATCAA | pYES2 |
| CBU1524 R2 | ACATGCATGCCTATGTCCTTTTGGGAGCGT | pYES2 |
| CBU1525 F2 | AAGGATCCAACACAATGATCAGCGATTTTACTTCGG | pYES2 |
| CBU1525 R2 | GCTCTAGATTAAATCGTAAGGCGCTTTGTT | pYES2 |
| CBU1532 F2 | AAGGATCCAACACAATGGCAGGCATAGCTGCAAC | pYES2 |
| CBU1532 R2 | ACATGCATGCTTACTTATTAAATTCGGGTATGTA | pYES2 |
| CBU1776 F2 | AAGGATCCAACACAATGTATATTAACATCAATCAATCC | pYES2 |
| CBU1776 R2 | GCTCTAGATTACTTTATAAAAGTCCGTGTATA | pYES2 |
| CBU1823 F2 | AAGGATCCAACACAATGCCTAAACTCAGTAACCGT | pYES2 |
| CBU1823 R2 | GCTCTAGATTATGGCCTCTTATTTGTTGGC | pYES2 |
| CBU1825 F2 | AAGGATCCAACACAATGTTGGTTAGTAATACCAGCA | pYES2 |
| CBU1825 R2 | GCTCTAGACTATTTTTTTGTCATTTCCAGATT | pYES2 |
| CBU1963 F2 | AAGGATCCAACACAATGGAGTTTCTAATAAAGTTTTCT | pYES2 |
| CBU1963 R2 | ACATGCATGCTCAAGGATGATGGTGGCGAG | pYES2 |
| CBU2052 F2 | AAGGATCCAACACAATGCCTAAAAACACAAATCCAG | pYES2 |
| CBU2052 R2 | GCTCTAGATTATTTCAAAAAAGCATTTACAAG | pYES2 |
| CBU2056 F2 | AAGGATCCAACACAATGAGTTTAATTTTCATAAAGATTC | pYES2 |
| CBU2056 R2 | GCTCTAGACAAGGTTAGAAGGAGCTTGAA | pYES2 |
| CBU2059 F2 | AAGGATCCAACACAATGTCGGAACCCCCGAATAA | pYES2 |
| CBU2059 R2 | GCTCTAGATCATTTGATTGTTAAGGAAGAAG | pYES2 |
| *icmL* F (1) | GCGCTCCTGTCACTGTCG | *dotI/icmL* mutant confirmation |
| *icmL* R (4) | GCGACCCCGTCTTGCCG | *dotI/icmL* mutant confirmation |
| Transposon (2) | GGTCGGAACAGGAGAGCG | *dotI/icmL* mutant confirmation |
| Transposon (3) | GGGTATGGAGAGGGATATTTC | *dotI/icmL* mutant confirmation |
| BlaM-0077 F | GGGAAGCGGTGTCGACATGAGACAACTCGTTTCAATTAAA | pJB-Cm:BlaM |
| BlaM-0077 R | GCATGCCTCAGTCGACTTACATAATAGAACACCCACGA | pJB-Cm:BlaM |
| BlaM-0635 F | GGGAAGCGGTGTCGACATGCGAGAGGAAAAAGAGGA | pJB-Cm:BlaM |
| BlaM-0635 R | GCATGCCTCAGTCGACCTAAACTAATGTCATTAAACGGT | pJB-Cm:BlaM |
| BlaM-1524 F | GGGAAGCGGTGTCGACATGAACACAAGTCCTACATCAA | pJB-Cm:BlaM |
| BlaM-1524 R | GCATGCCTCAGTCGACCTATGTCCTTTTGGGAGCGT | pJB-Cm:BlaM |
| *icmL.1* F | ATGACCAAGACAAGTGAAGGG | *icmL.1* probe |
| *icmL.1* R | TTACACCGAATTATCTACCGCA | *icmL.1* probe |
| TnF | ATGCACCCAGAAACGCTGG | Tn probe |
| TnR | CCAATGCTTAATCAGTGAGG | Tn probe |
| icmQ In-Fusion F | CCTTCATGAAGGAGGCTGCAGTTCACCAAAAGGGCTGGGATT | p*QC* |
| IcmC In-Fusion R | GCATGCCTCAGTCGACCTAAACCGACATTCCTAACACT | p*QC* |
| icmL In-Fusion R | CCTTCATGAAGGAGGCTGCAGTAGCGCGGTTAGGGGATAAC | p*icmL* |
| icmL In-Fusion R | GCATGCCTCAGTCGACTTACACCGAATTATCTACCGCA | p*icmL* |
